# Human hippocampal theta–gamma coupling coordinates sequential planning during navigation

**DOI:** 10.1073/pnas.2513547123

**Published:** 2026-02-27

**Authors:** Zimo Huang, James A. Bisby, Neil Burgess, Daniel Bush

**Affiliations:** ^a^Department of Neuroscience, Physiology and Pharmacology, University College London, London WC1E 6BT, United Kingdom; ^b^Division of Psychiatry, University College London, London W1T 7BN, United Kingdom; ^c^UCL Queen Square Institute of Neurology, University College London, London WC1N 3BG, United Kingdom; ^d^UCL Institute of Cognitive Neuroscience, University College London, London WC1N 3AZ, United Kingdom; ^e^Wellcome Centre for Human Neuroimaging, University College London, London WC1N 3AR, United Kingdom

**Keywords:** hippocampus, navigation, neural coding, phase-amplitude coupling, MEG

## Abstract

Human behavior often relies on executing a specific sequence of actions to achieve a desired outcome. However, the neural mechanisms underlying the dynamic construction and maintenance of such sequences during goal-directed behavior are not yet clear. Empirical and theoretical studies of working memory function suggest that sequential information may be encoded in neural circuits by bursts of gamma activity occurring at consecutive theta phases. Here, we asked whether a similar coding scheme might support sequential planning during goal-directed navigation. Using noninvasive magnetoencephalography and an abstract navigation task, we found that hippocampal theta power during both planning and subsequent navigation decreased with proximity to the current goal, only during accurate navigation. At the same time, theta–gamma phase–amplitude coupling (PAC) increased with goal proximity, consistent with sequences of upcoming locations being represented by gamma bursts occurring at successive theta phases. Importantly, entorhinal high gamma and hippocampal low gamma dominated while traversing novel and previously experienced paths, respectively, consistent with previous rodent studies. These findings suggest that hippocampal theta–gamma PAC flexibly and dynamically coordinates sequences of actions during goal-directed behavior across mammalian species, using different gamma bands for mnemonic and prospective planning.

## Significance

Human behavior often relies on executing specific sequences of actions to achieve desired outcomes—like planning the series of locations one needs to pass through to reach a given destination. However, the neural mechanisms underlying sequential planning are currently unclear. Here, we show that neural activity during navigation is consistent with sequences of upcoming locations being coded within bursts of high frequency gamma activity occurring at successive phases of a lower frequency theta oscillation. This coding scheme has previously been proposed to support short-term memory function and is observed in the rodent brain during active navigation. Hence, our results suggest that this “phase–amplitude coupling” may be a general mechanism for encoding sequential information across cognitive domains and mammalian species.

Human behavior often relies on executing a specific sequence of actions to achieve a desired outcome. However, the neural mechanisms underlying the dynamic construction and maintenance of such sequences during goal-directed behavior are not yet clear. Empirical and theoretical studies of working memory function suggest that sequential information may be encoded in neural circuits using theta–gamma phase–amplitude coupling (PAC) (1, 2). According to such a scheme, successive items are represented by bursts of gamma activity at successive theta phases. This framework predicts both an increase in theta power and a decrease in PAC with sequence length, as gamma activity becomes more distributed across the theta cycle (3). Each of these predictions have been validated in empirical studies of working memory function, showing that theta power in the frontal lobe increases (4) and PAC in the medial temporal lobe decreases (5, 6) with memory load. We hypothesized that the same neural mechanisms might be used to support the flexible planning, maintenance, and execution of action sequences during goal-directed behavior.

A canonical example of sequential planning comes from spatial navigation, where specific routes through known locations are constructed and maintained in memory while being dynamically executed. This form of navigation is believed to rely on a ‘cognitive map’ that is supported by the mammalian hippocampus (7). In rodents, the hippocampal local field potential (LFP) is dominated by theta oscillations during movement (8), and increased theta band activity is also observed in the human hippocampus during navigation in virtual (9) and real-world (10, 11) environments. Intriguingly, increases in theta power are often greatest immediately prior to the onset of movement (12–17), and movement-related theta power correlates with distance to the current goal both prior to (13, 18) and during (19) virtual navigation. In the rodent hippocampus, theta oscillations have also been shown to modulate gamma power in two distinct bands: a slower band, generated locally and implicated in memory retrieval; and a faster band, originating in the entorhinal cortex and implicated in the encoding of new information (20). In addition, hippocampal place cells fire at progressively earlier phases of each theta cycle as their firing fields are traversed (21). This can generate ‘theta sweeps’ of place cell activity in each oscillatory cycle that encode upcoming spatial trajectories toward current goals (22–26).

Here, we asked whether theta–gamma PAC might support the flexible construction and dynamic maintenance of action sequences during goal-directed navigation. Specifically, we used magnetoencephalography (MEG) to assay whole-brain activity while participants performed a self-paced navigation task. In this task, participants must learn the spatial layout of an abstract map of images while planning routes to specific goals within that map. First, we found that hippocampal theta power during spatial planning correlates with distance to the goal, only during trials in which a direct path to the goal is known. In addition, we observed a dynamic reduction in hippocampal theta power during subsequent navigation, as participants approached the goal. Second, we found that theta–gamma PAC increases dynamically as participants approach the goal during navigation. Importantly, theta phase modulates fast gamma amplitude in the entorhinal cortex during learning, when participants traverse novel routes; and slow gamma amplitude in the hippocampus during subsequent retrieval, when participants retrace previously experienced paths, consistent with rodent data (20). These findings support the hypothesis that hippocampal theta–gamma phase–amplitude coupling supports the planning, maintenance, and execution of action sequences during navigation, providing insight into the neural mechanisms of goal-directed behavior across mammalian species.

## Result

### Behavioral Data.

**Fig. 1.**
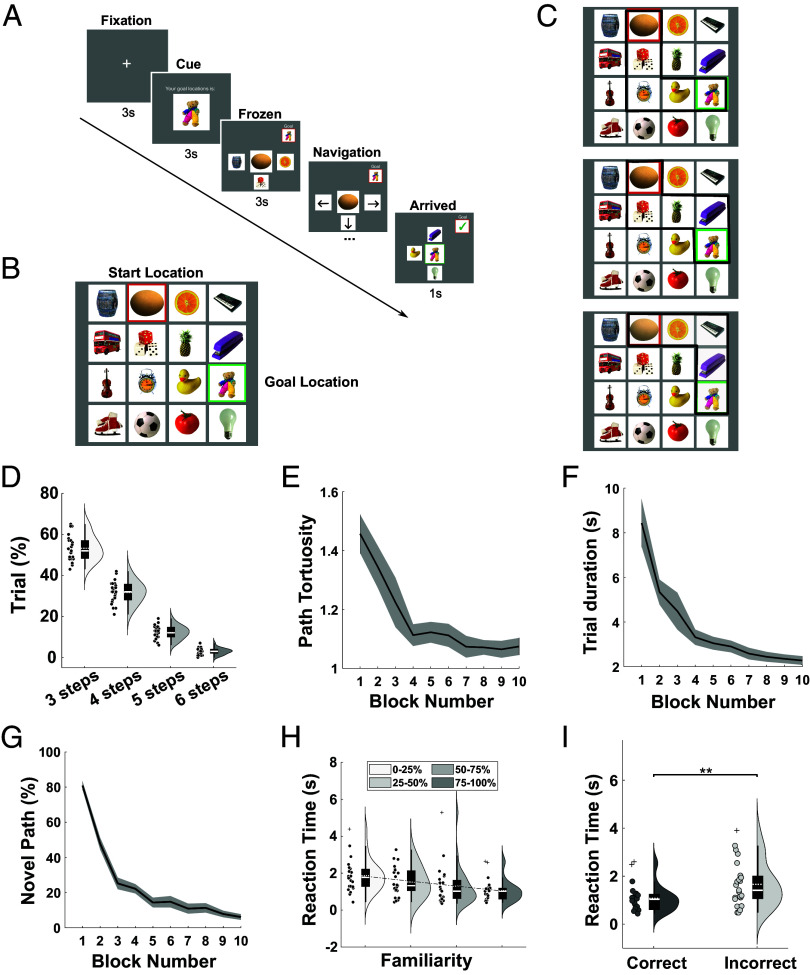
Schematic illustration of task structure and behavioral data. (*A*) During each trial, participants were cued with their goal location for 3 s (“cue period”) after a 3 s fixation period and then frozen at their starting location on the abstract image map for 3 s (“frozen period”) before being allowed to navigate freely. During navigation, participants could use four buttons to move directly up, down, left, or right from their current location on the abstract image map. Participants were instructed to find the shortest path from their current location to the given goal location in each trial. (*B*) Example abstract image map composed of 16 visual stimuli. (*C*) A subset of the potential shortest paths between start and goal locations shown in (*B*). There are often multiple shortest paths available between a given start and goal location. (*D*) Distribution of shortest possible path distance from start to goal across 100 trials per participant. (*E*) Changes in path tortuosity across blocks of 10 trials. (*F*) Changes in time taken to complete each path by block. (*G*) Changes in the proportion of completely novel paths traversed by block. (*H*) Reaction time taken to make the first transition, binned by the proportion of transitions that were previously experienced on the subsequent path. (*I*) Reaction times for correct and incorrect trials. Box plots show mean (dashed) and median (solid line), *Lower* and *Upper* quartiles (*Top* and *Bottom* of box), minimum and maximum values (excluding outliers, *Top* and *Bottom* whiskers) across participants unless otherwise stated. **P < 0.01.

We asked 23 participants to complete an abstract navigation task inside the MEG scanner. In this task, participants moved across a fixed 4 × 4 grid of images to find a specific goal image in each trial using as few steps (i.e., button presses) as possible (Fig. 1 *A–D*). Participants rapidly learned the map layout and could subsequently navigate efficiently from start to goal locations in each trial. In particular, both path tortuosity (the total number of steps taken divided by the shortest possible path, Fig. 1*E*) and trial duration (time taken to reach the goal during navigation, Fig. 1*F*) decreased rapidly over the course of the task as participants came to experience every possible transition on the map (Fig. 1*G*). The time taken to begin moving across the map also decreased with familiarity of the upcoming path (Fig. 1*H*), suggesting that participants took less time to plan familiar routes [t(22) = −6.04, *P* < 0.001, one-sample *t* test, Cohen’s d = 1.22]. Similarly, the time taken to execute each button press during navigation decreased with prior experience of each transition [t(22) = −7.06, *P* < 0.001, one-sample *t* test, Cohen’s d = 1.42]. In total, participants took the “correct” (i.e., shortest) path in 86.7 ± 2.18% of trials, and reaction times in those trials were slightly but significantly faster than in incorrect trials [t(22) = −3.71, *P* = 0.0012, paired *t* test, Cohen’s d = 0.725; Fig. 1*I*]. These results demonstrate that participants rapidly learned the map structure and could accurately plan and execute both novel and familiar paths to given goal locations.

### Hippocampal Theta Power during Planning Encodes Goal Distance.

**Fig. 2.**
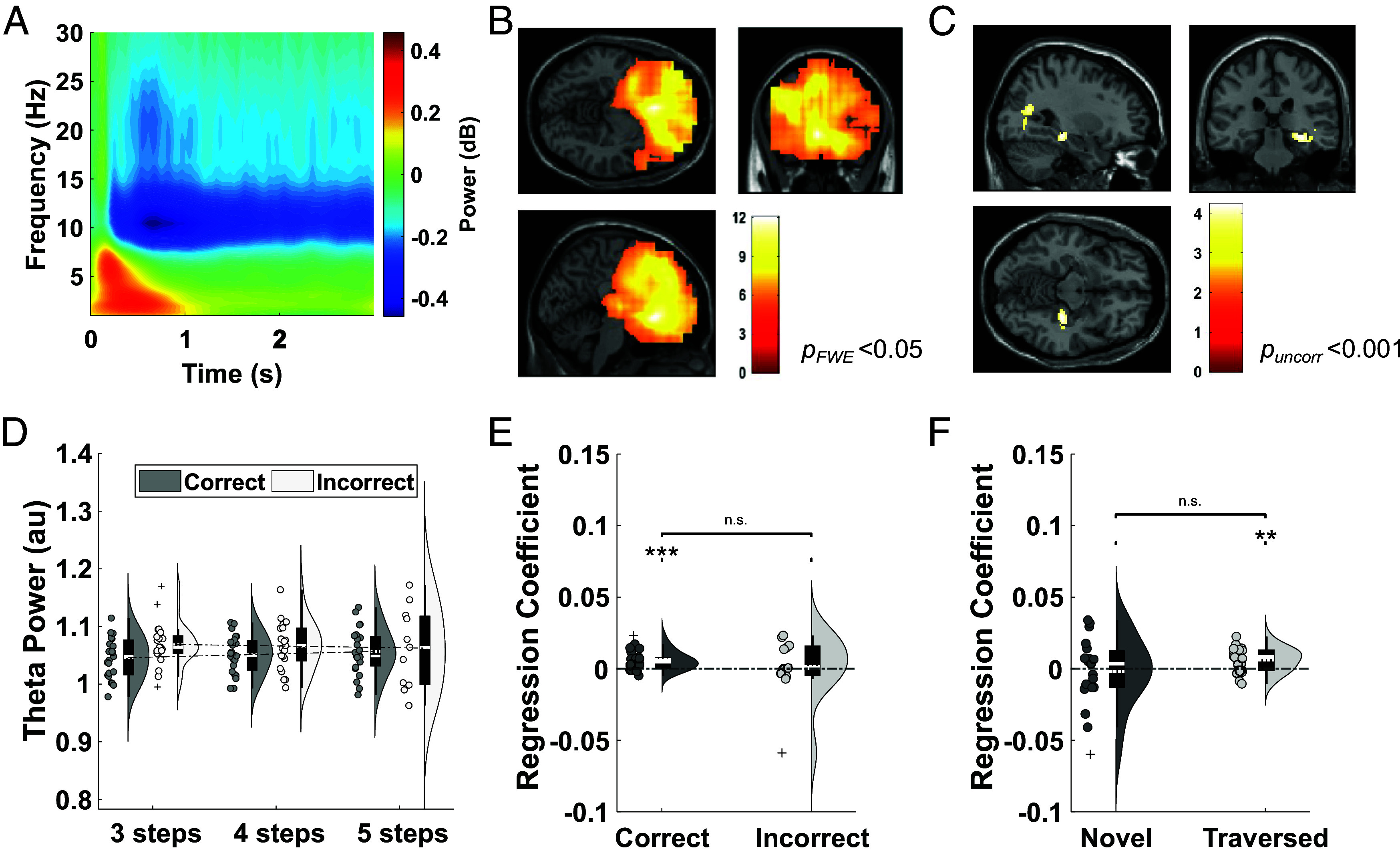
Theta power covaries with distance to the goal during planning. (*A*) Spectrogram of low-frequency oscillatory power during the 3 s cue period, baseline corrected by mean power during 1 s of the preceding fixation period. (*B*) Source localization of 2 to 5 Hz theta power during the 3 s cue period (visualized at *p_FWE_* < 0.05). The largest cluster showing a significant increase in theta power is centered on the frontal cortex [(−4, 24, −8); Z = 6.6, *p_FWE <_* 0.001]. (*C*) Source localization of 2 to 5 Hz theta power during the 3 s cue period that covaries positively with the shortest path distance to the goal (visualized at *p_uncorr_* < 0.001). (*D*) Mean 2 to 5 Hz theta activity extracted from a predefined right hippocampal ROI across the 3 s cue period for trials with different shortest path lengths, shown separately for correct and incorrect trials. (*E*) Mean regression coefficient of 2 to 5 Hz theta activity extracted from the right hippocampal ROI against distance to goal in correct [t(22) = 4.11, *P* < 0.001, one-sample *t* test, Cohen’s d = 0.827] and incorrect [t(22) = 0.203, *P* = 0.843, one-sample *t* test, Cohen’s d = 0.057] trials. No significant difference is observed between correct and incorrect trials [t(22) = 0.713, *P* = 0.492, paired *t* test, Cohen’s d = 0.295]. (*F*) Mean regression coefficient of 2 to 5 Hz theta activity extracted from the right hippocampal mask against distance to goal for the novel [t(22) = −0.362, *P* = 0.722, one-sample *t* test, Cohen’s d = 0.080] and previously traversed [t(22) = 3.44, *P* = 0.0023, one-sample *t* test, Cohen’s d = 0.692] paths. No significant difference is observed between novel and traversed trials [t(22) = −1.52, *P* = 0.148, paired *t* test, Cohen’s d = 0.466]. The color bar in panels (*B* and *C*) shows t-statistics, and a list of all significant clusters can be found in *SI Appendix*, Table S1. Box plots show mean (dashed) and median (solid line), *Lower* and *Upper* quartiles (*Top* and *Bottom* of box), minimum and maximum values (excluding outliers, *Top* and *Bottom* whiskers) across participants unless otherwise stated. **P < 0.01, ***P < 0.001.

Theoretical models of working memory maintenance by theta–gamma phase–amplitude coupling suggest that theta power should increase linearly with sequence length (1–3). Consistent with this hypothesis, human frontal theta power has been shown to increase with working memory load (4). Similarly, previous intracranial electroencephalography (EEG) studies have demonstrated that theta power in the human hippocampal formation increases with distance to a hidden goal during navigation (13, 19). To establish whether the same neural mechanisms might support sequential planning during goal-directed navigation, we examined the spectral content of the MEG signal collapsed across all sensors during the 3 s cue period (when the goal location for that trial was displayed on screen). We focused our analyses on a 2 to 5 Hz theta frequency band that has previously been implicated in human spatial cognition (13, 18, 19, 27). We observed an increase in 2 to 5 Hz theta power relative to a preceding baseline period that peaked during the first 1 s (Fig. 2*A*). To identify the source of this signal, we source localized 2 to 5 Hz theta power during the full 3 s window and found a large cluster centered on the prefrontal cortex [(−4, 24, −8); Z = 6.60, *p*_FWE_ = 0.005, Fig. 2*B*]that extended into the bilateral medial temporal lobes [(6, −18, −44); Z = 4.34, *p*_FWE_ = 0.022; *SI Appendix*, Fig. S1*A*]and hippocampi [(−32, −20, −20), Z = 4.74, *p_FWE, SVC_* < 0.05; *SI Appendix*, Fig. S1*B*].

Next, to examine whether this oscillatory signal might support the planning of upcoming spatial trajectories, we covaried theta power with step distance to the goal location during “correct” trials (where participants took the shortest path to the goal), separately for each source voxel. This revealed a significant cluster in the right medial temporal lobe [(30, −28, 10); Z = 3.59, *p_uncorr_* < 0.001, Fig. 2*C*]which passed our cluster-level statistical threshold within a predefined right hippocampal ROI [(30, −28, −10); Z = 3.59, *p_FWE, SVC_* = 0.006; *SI Appendix*, Fig. S1*C*; see *SI Appendix*, Table S1 for a list of all clusters identified by source localization analyses]. No significant clusters were identified when the analysis was repeated using data from incorrect trials, either on a whole-brain level or within the same right hippocampal ROI. To further explore this effect, we extracted average theta power from the predefined right hippocampal ROI separately for correct and incorrect trials with 3, 4, and 5-step shortest paths to the goal (Fig. 2*D*). This illustrates a significant decrease in theta power with shortest path length from start to goal locations for correct trials [t(22) = 4.11, *P* < 0.001, one-sample *t* test, Cohen’s d = 0.827; *SI Appendix*, Fig. S1*D*], but no overall change for incorrect trials (where the participants did not know the shortest path to the goal, Fig. 2*E*). This is consistent with our hypothesis that the planning of upcoming paths is supported by hippocampal theta–gamma phase–amplitude coupling, whereby longer sequences elicit greater theta power as gamma activity becomes more distributed across each low frequency cycle. Importantly, this effect was not driven by changes in the aperiodic slope of the power spectrum (*SI Appendix*, Fig. S2*A*) or theta band amplitude of the evoked response (*SI Appendix*, Fig. S2*B*). Moreover, in contrast to previous studies (19), we found no evidence for a difference in the strength of theta power modulation by goal distance between the anterior and posterior hippocampus (*SI Appendix*, Fig. S3*A*).

Next, we aimed to establish whether the coding of goal distance in theta band oscillations arose equally from the planning of novel routes and the retrieval of previously executed paths. To do so, we performed the same linear regression analysis between trial-by-trial theta power extracted from the right hippocampal ROI and distance to the goal, splitting correct trials into novel paths (where at least one transition had not been experienced before, in either direction) and previously traversed paths (where all transitions had been experienced before). Interestingly, a significant relationship between theta activity and goal distance was only observed for previously traversed paths [t(22) = 3.44, *P* = 0.0023, one-sample *t* test, Cohen’s d = 0.692; Fig. 2*F*]. This suggests that the theta power code for goal distance during spatial planning primarily reflects the retrieval of previously experienced navigational trajectories.

### Hippocampal Theta Power during Navigation Encodes Goal Distance.

**Fig. 3.**
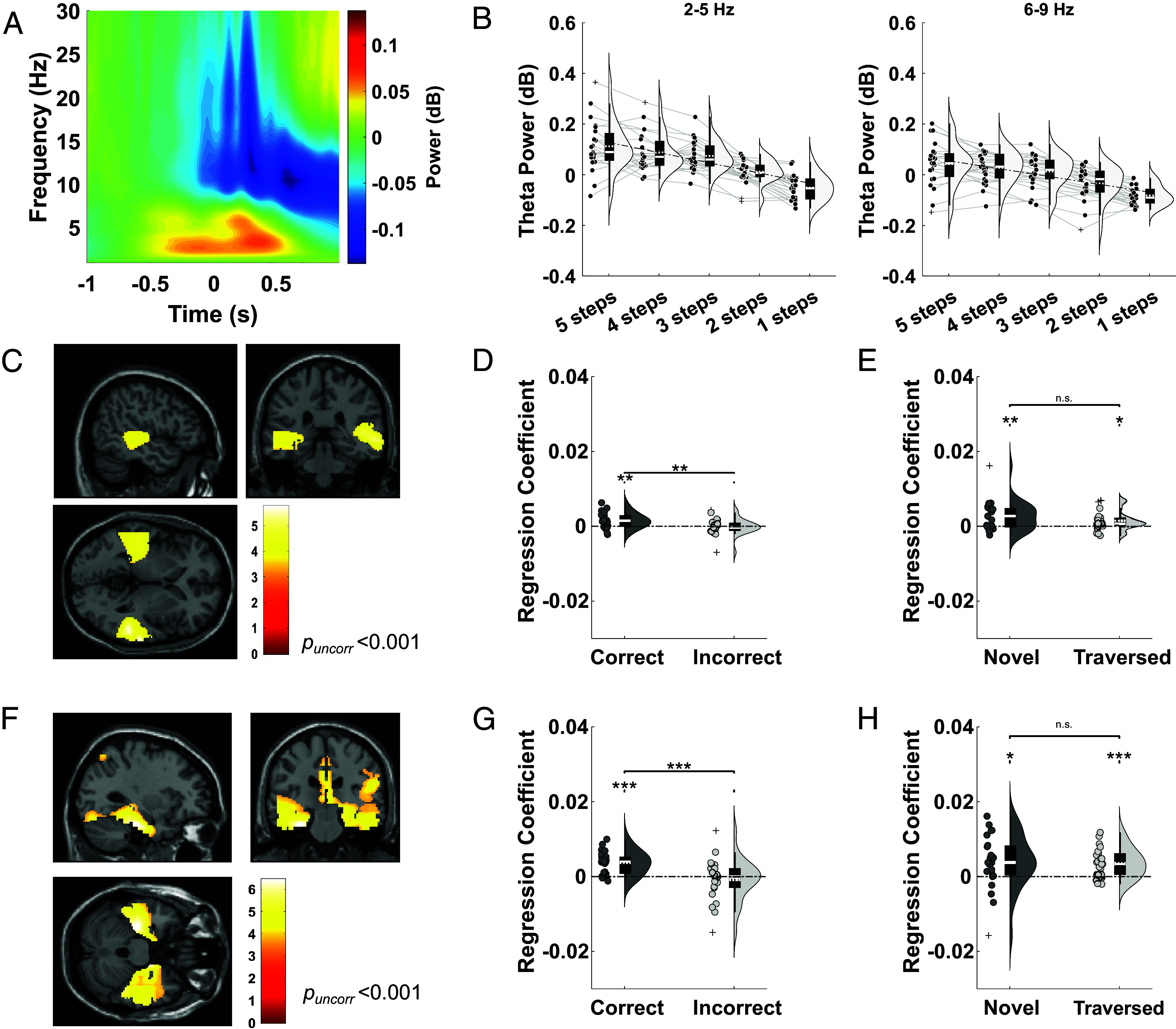
Theta power covaries with distance to the goal during navigation. (*A*) Spectrogram of low-frequency oscillatory power during 2 s epochs centered on each button press during navigation, baseline corrected by mean power during the first 0.5 s of the 2 s epoch around each button press. (*B*) Average 2 to 5 Hz and 6 to 9 Hz theta power across all sensors for correct trials, split by distance to the goal in descending order during navigation. (*C*) Source localization of the 2 to 5 Hz theta power vs. goal distance relationship (visualized at *p_uncorr_* < 0.001 without masking). Two clusters passed our cluster level significance threshold within a bilateral hippocampal mask [*Left*: (−34, −34, −2); Z = 3.43; *p_FWE, SVC_* = 0.017; *Right*: (36, −14, −24); Z = 3.14, *p_FWE, SVC_* = 0.041]. (*D*) Mean regression coefficients for average 2 to 5 Hz theta power extracted from a bilateral hippocampal mask against distance to the goal for correct and incorrect trials. (*E*) Mean regression coefficients for average 2 to 5 Hz theta power extracted from a bilateral hippocampal mask against distance to the goal for novel and traversed trials. No significant difference is observed [t(22) = 1.34, *P* = 0.195, paired *t* test, Cohen’s d = 0.364]. (*F*) Source localization of the 6 to 9 Hz theta power vs. goal distance relationship (visualized at *p_uncorr_* < 0.001 without masking). Two clusters passed our cluster-level significance threshold within a bilateral hippocampal mask [*Left*: (−34, −26, −16); Z = 4.15; *p_FWE, SVC_* < 0.001; *Right*: (16, −10, −18); Z = 4.47; *p_FWE, SVC_* < 0.001]. (*G*) Mean regression coefficients for average 6 to 9 Hz theta activity extracted from a bilateral hippocampal mask against distance to the goal for correct and incorrect trials. (*H*) Mean regression coefficients for average 6 to 9 Hz theta power extracted from a bilateral hippocampal mask against distance to the goal for novel and traversed paths. No significant difference is observed [t(22) = 0.127, *P* = 0.90, paired *t* test, Cohen’s d = 0.0376]. The color bar in panels (*C* and *F*) shows t-statistics, and a list of all significant clusters can be found in *SI Appendix*, Table S1. Box plots show mean (dashed) and median (solid line), *Lower* and *Upper* quartiles (*Top* and *Bottom* of box), minimum and maximum values (excluding outliers, *Top* and *Bottom* whiskers) across participants unless otherwise stated. *P < 0.05, **P < 0.01, ***P < 0.001.

Next, we asked whether the relationship between theta power and distance to the goal would persist throughout navigation, as suggested by previous intracranial EEG studies (19). To do so, we first examined changes in oscillatory power averaged across all MEG sensors on a movement-by-movement basis as participants navigated across the map. We observed an increase in theta power in a 2 s window centered on each button press compared with a baseline period when participants were stationary on the map that peaked in a ~1 s window around translational movement (Fig. 3*A*). Next, following previous studies of virtual navigation (13), we extracted average power in low (2 to 5 Hz) and high (6 to 9 Hz) theta bands across all sensors during the 2 s period around each button press during navigation (excluding the final step, when the goal is visible on screen). For correct trials (where participants knew where they were going), average power across all sensors in both low [t(22) = 8.28, *P* < 0.001, one-sample *t* test, Cohen’s d = 1.67] and high [t(22) = 10.19, *P* < 0.001, one-sample *t* test, Cohen’s d = 2.05] theta bands iteratively decreased as the goal was approached, but this effect was less consistent for incorrect trials, where participants failed to take their shortest route to the goal (Fig. 3*B* and *SI Appendix*, Fig. S4).

To identify the source of this signal, we covaried step distance to the goal location during “correct” trials (where the participants knew the shortest path to reach the goal) with theta power in each source voxel. This revealed two significant clusters centered over the bilateral temporal lobes for both low [(52, −38, 4); Z = 4.45, *p_FWE_* = 0.017, Fig. 3*C*] and high [(−30, −24, −26); Z = 4.77, *p_FWE_* = 0.005; Fig. 3*F*] theta bands, each of which passed our cluster level statistical threshold within a predefined bilateral hippocampal ROI [low theta: (−34, −34, −2); Z = 3.43, *p_FWE, SVC_* < 0.05; *SI Appendix*, Fig. S5*A*; high theta: (16, −10, −18); Z = 4.47, *p_FWE, SVC_* < 0.05; *SI Appendix*, Fig. S6*A*]. To further explore this effect, we extracted average theta power from the same bilateral hippocampal ROI separately for correct and incorrect trials with different numbers of steps remaining to the goal location. For correct trials, we found a significant positive relationship between theta power in the bilateral hippocampus and distance remaining to the goal in both the 2 to 5 Hz [t(22) = 3.51, *P* = 0.002, one-sample *t* test, Cohen’s d = 0.707; Fig. 3*D* and *SI Appendix*, Fig. S5*B*] and 6 to 9 Hz [t(22) = 5.71, *P* < 0.001, one-sample *t* test, Cohen’s d = 1.15; Fig. 3*G* and *SI Appendix*, Fig. S6*B*] frequency bands. In both cases, theta power decreased iteratively as the goal was approached in correct trials only (*SI Appendix*, Fig. S5 *C*–*E* and S6*C*), and the relationship between theta power and distance to the goal was stronger in correct vs. incorrect trials [low theta: t(22) = 3.37, *P* = 0.0028, paired *t* test, Cohen’s d = 0.730; Fig. 3*D*; high theta: t(22) = 4.10, *P* < 0.001, paired *t* test, Cohen’s d = 0.949; Fig. 3*G*]. Again, we confirmed that this effect was not driven by changes in the aperiodic slope of the power spectrum (*SI Appendix*, Fig. S2*C*) or theta band amplitude of the evoked response (*SI Appendix*, Fig. S2 *D* and *E*) and found no differences in the strength of theta modulation by goal distance between the anterior and posterior hippocampus (*SI Appendix*, Fig. S3*B*).

Finally, we sought to confirm whether the theta power code for goal distance arose primarily during the navigation of previously traversed routes, as observed during the cue period. During navigation, however, we observed a significant positive relationship between bilateral hippocampal 2 to 5 Hz theta power and distance remaining to the goal for both novel [t(22) = 3.31, *P* = 0.0032, one-sample *t* test, Cohen’s d = 0.667] and previously traversed paths [t(22) = 2.72, *P* = 0.0124, one-sample *t* test, Cohen’s d = 0.548; Fig. 3*E*]. Similarly, a significant positive relationship between bilateral hippocampal 6 to 9 Hz theta power and distance to the goal was present for both novel [t(22) = 2.55, *P* = 0.0181, one-sample *t* test, Cohen’s d = 0.514] and previously traversed paths [t(22) = 4.49, *P* < 0.001, one-sample *t* test, Cohen’s d = 0.904; Fig. 3*H*]. This suggests that hippocampal theta power coding of upcoming spatial trajectories during active navigation does not depend on prior experience of the route being traversed.

### Theta–Gamma PAC Mediates Spatial Planning.

Next, we asked whether the phase of theta oscillations modulated the amplitude of concurrent gamma band activity to organize sequential planning during navigation. According to this hypothesis, the encoding of longer sequences corresponds to a wider theta phase distribution of gamma power, which should therefore be negatively correlated with the strength of theta–gamma PAC (TG-PAC) (5, 6). Hence, we examined whether TG-PAC increased as the goal was approached and an increasingly short sequence of upcoming locations needed to be maintained in memory. We focused our analyses on low (30 to 70 Hz) and high (70 to 140 Hz) gamma bands that have been shown to exhibit distinct functional roles in previous studies of rodent (20) and human (6, 28) memory function.

**Fig. 4.**
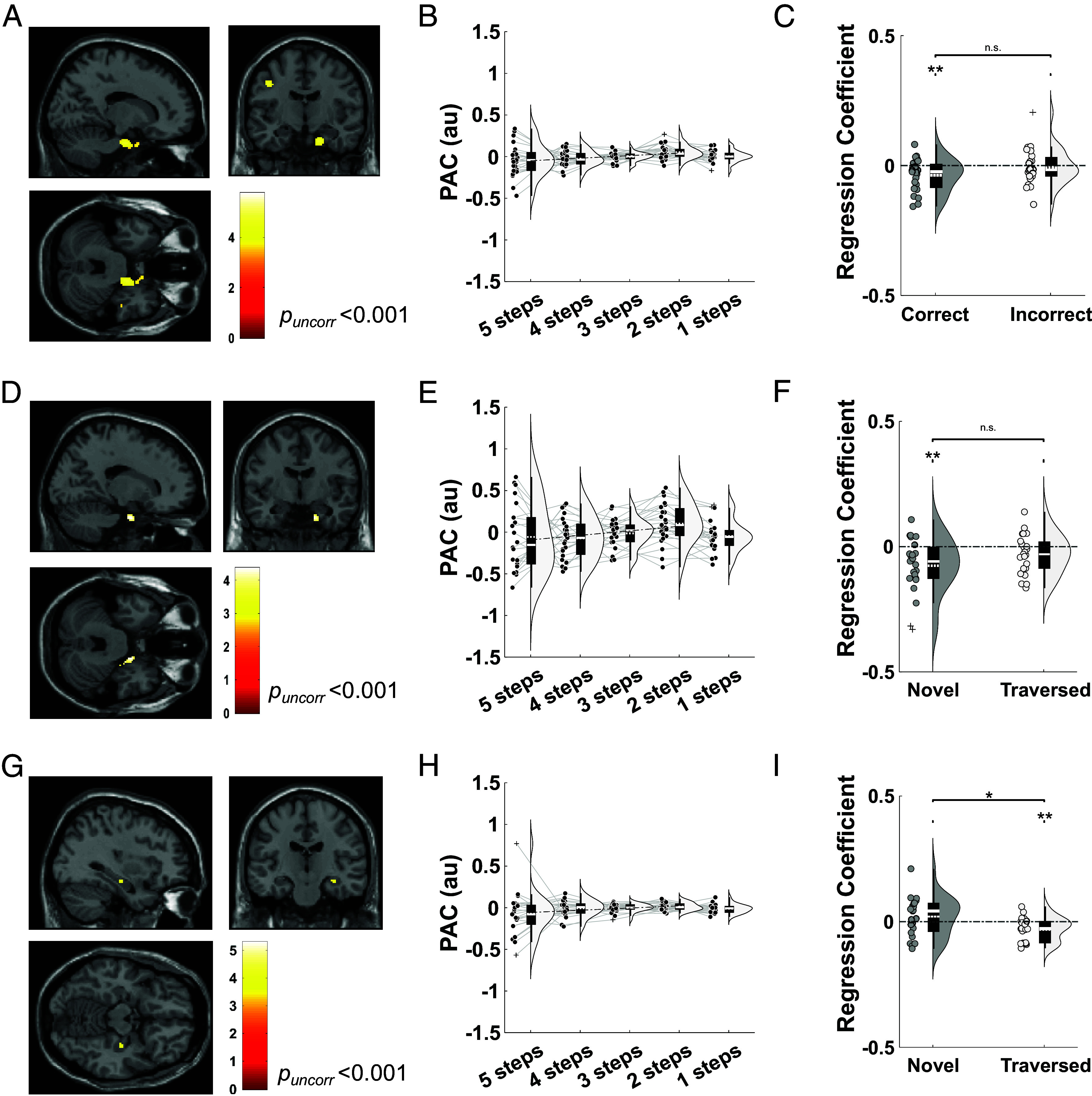
Theta–gamma phase–amplitude coupling covaries with distance to the goal during navigation. (*A*) Source localization of the 2 to 5 Hz theta—70 to 140 Hz fast gamma phase–amplitude coupling vs. goal distance relationship for all correct trials (visualized at *p_uncorr_* < 0.001). A significant cluster is observed in the right anterior temporal lobe. (*B*) Average theta-fast gamma PAC extracted from a predefined entorhinal mask for all correct trials, split by distance to the goal in descending order during navigation. (*C*) Regression coefficients between theta-fast gamma PAC and goal distance in the right entorhinal mask for correct and incorrect trials (excluding the final step, when the goal is visible on screen). No significant difference is observed [t(22) = −1.65, *P* = 0.11, paired *t* test, Cohen’s d = 0.472]. (*D*) Source localization of the theta–fast gamma PAC vs. goal distance relationship for all correct trials that use novel paths (visualized at *p_uncorr_* < 0.001). (*E*) Average theta-fast gamma PAC extracted from a predefined right entorhinal mask for all correct trials that use novel paths, split by distance to the goal in descending order during navigation. (*F*) Regression coefficients between theta-fast gamma PAC and goal distance in the right entorhinal mask, for all correct trials that use novel and previously traversed paths (excluding the final step, when the goal is visible on screen). No significant difference is observed [t(22) = −1.62, *P* = 0.119, paired *t* test, Cohen’s d = 0.459]. (*G*) Source localization of the 2 to 5 Hz theta—30 to 70 Hz slow gamma phase–amplitude coupling vs. goal distance relationship for all correct trials that use previously traversed paths (visualized at *p_uncorr_* < 0.001). (*H*) Average theta-slow gamma PAC extracted from a predefined right hippocampal mask for all correct trials that use previously traversed paths, split by distance to the goal in descending order during navigation. (*I*) Regression coefficients between theta-slow gamma PAC and goal distance in the right hippocampal mask for all correct trials that use novel and previously traversed paths (excluding the final step, when the goal is visible on screen). The color bar in panels (*A*, *D*, and *G*) shows t-statistics. Box plots show mean (dashed) and median (solid line), *Lower* and *Upper* quartiles (*Top* and *Bottom* of box), minimum and maximum values (excluding outliers, *Top* and *Bottom* whiskers) across participants unless otherwise stated. *P < 0.05, **P < 0.01.

First, we examined dynamic changes in TG-PAC between 2 to 5 Hz theta phase and 70 to 140 Hz fast gamma amplitude as the goal is approached during navigation. To do so, we covaried step distance to the goal with TG-PAC in each source space voxel (excluding the final step, when the goal is visible on screen). This revealed a significant cluster in the right anterior temporal lobe for correct trials [(18, −8, −30); Z = 3.89, *p_uncorr_* < 0.001, Fig. 4*A*], which passed our cluster level statistical threshold in a predefined right entorhinal cortex ROI [(18, −10, −30); Z = 3.91, *p_FWE, SVC_* = 0.004; *SI Appendix*, Fig. S7*A*]. To further explore this effect, we extracted estimates of TG-PAC from the right entorhinal ROI and conducted a linear regression against distance remaining to the goal. We observed the same negative correlation between TG-PAC and distance to the goal in correct [t(22) = −2.91, *P* = 0.0082, one-sample *t* test, Cohen’s d = 0.585] but not incorrect [t(22) = −0.340, *P* = 0.737, one-sample *t* test, Cohen’s d = 0.0684] trials (Fig. 4 *B* and *C* and *SI Appendix*, Fig S7*B*). Intriguingly, we found that this relationship between theta–fast gamma phase–amplitude coupling and distance to the goal during navigation was present for novel [t(22) = −3.30, *P* = 0.0032, one-sample *t* test, Cohen’s d = 0.665; Fig. 4 *D*–*F*) but not previously traversed paths [t(22) = −1.88, *P* = 0.0729, one-sample *t* test, Cohen’s d = 0.379]. Consistent with this, covarying step distance to the goal during novel trials with TG-PAC in each source space voxel revealed a significant cluster in the right entorhinal cortex [(18, −6, −30); Z = 3.65, *p_uncorr_*< 0.001] which passed our cluster-level statistical threshold in a predefined right entorhinal cortex ROI [(22, −18, −30); Z = 3.77, *p_FWE, SVC_* = 0.006; *SI Appendix*, Fig. S7*C*]. This is consistent with previous rodent electrophysiology studies that describe increased phase–amplitude coupling between theta and fast gamma band activity originating in the entorhinal cortex during the encoding of new information (20). Interestingly, similar effects were observed during the cue period (*SI Appendix*, Fig. S8). Importantly, in both cases, the relationship between TG-PAC and goal distance did not arise spuriously from changes in theta cycle asymmetry (*SI Appendix*, Fig. S9).

Next, we examined TG-PAC between 2 to 5 Hz theta phase and 30 to 70 Hz slow gamma amplitude. Although we did not observe any significant clusters at the whole brain level when including all correct trials (*SI Appendix*, Fig. S10*A*), we identified a cluster in the right hippocampus that showed a negative relationship between TG-PAC and distance to the goal during the traversal of previously experienced paths only ([34, −18, −14]; Z = 3.25, *p_uncorr_* = 0.001; Fig. 4*G*), which shows a trend toward significance in a predefined right hippocampal ROI [(34, −18, −14); Z = 3.25, *p_FWE, SVC_* = 0.051; *SI Appendix*, Fig. S10*B*]. To further explore this effect, we conducted the same regression analysis between trial-by-trial estimates of TG-PAC obtained from the right hippocampal ROI and distance to the goal (Fig. 4*H*). This showed a significant negative relationship for previously traversed paths [t(22) = −3.07, *P* = 0.0056, one-sample *t* test, Cohen’s d = 0.618] but not for novel paths [t(22) = 1.25, *P* = 0.22, one-sample *t* test, Cohen’s d = 0.252], with the former being significantly stronger than the latter [t(22) = −2.26, *P* = 0.0342, paired *t* test, Cohen’s d = 0.751, Fig. 4*I* and *SI Appendix*, Fig. S10*C*]. This is consistent with previous rodent electrophysiology studies that describe increased phase–amplitude coupling between theta and slow gamma band activity originating within the hippocampus during the sequential reactivation of previously learned information (20).

In summary, TG-PAC in the hippocampal formation increases dynamically as a hidden goal is approached during navigation. This is consistent with the hypothesis that maintaining a longer sequence of items in memory—i.e., a greater number of locations that remain to be traversed—corresponds to a wider distribution of gamma power in each theta cycle and, therefore, lower TG-PAC (5, 6). In addition, we found that distinct fast and slow gamma bands originating in the entorhinal cortex and hippocampus support the prospective planning of new paths and the retrieval of previously traversed paths, respectively.

## Discussion

We have shown that human hippocampal theta power during both planning (immediately prior to movement onset) and navigation correlates with the distance to a hidden goal location, only in correct trials when participants are aware of the distance they need to travel. This replicates previous intracranial electrophysiology studies in epilepsy patients (13, 19) and extends those findings to a healthy population navigating in an abstract state space. Moreover, these results are analogous to fMRI studies showing that the hippocampal BOLD signal during navigation in naturalistic virtual environments correlates with path distance to a hidden goal, while the entorhinal BOLD signal correlates with Euclidean distance to the same goal (29). During the planning period, we also observed widespread increases in theta power across the frontal cortex that did not covary with subsequent path length. Alongside a role in cognitive control and working memory function (30), frontal midline theta oscillations have been implicated in spatial and episodic memory retrieval processes (31–33). Interestingly, the modulation of hippocampal theta power by subsequent path distance during this period was strongest prior to the traversal of previously experienced paths. Hence, our results suggest that the frontal theta rhythm coordinates the retrieval of past experience during spatial planning to provide an estimate of goal distance that is subsequently reflected in hippocampal theta power.

Our findings are consistent with theoretical models of working memory function which suggest that sequential information is encoded in neural circuits using theta–gamma phase–amplitude coupling (1, 2). Those models predict an increase in theta power and a decrease in phase–amplitude coupling during the maintenance of longer sequences, as gamma activity (and associated neural firing) becomes more widely dispersed across each theta cycle (3). Consistent with this, previous working memory studies have shown that frontotemporal theta power increases (4, 34, 35) and temporal lobe theta–gamma phase–amplitude coupling decreases (5, 6) with the number of items that must be actively maintained. Our results suggest that the same neural coding schemes used to passively encode sequential information during working memory function are also used to flexibly construct and maintain sequences of intended actions during spatial navigation. This would explain the dynamic reduction in hippocampal theta power and increase in hippocampal in theta–gamma phase–amplitude coupling as the goal is approached during navigation, and fewer locations remained to be traversed. In our task, it is unlikely that a working memory strategy was employed, given that the goal location was chosen randomly on each trial (preventing any rehearsal of the upcoming path); and there were a total of almost six hundred shortest paths between over a hundred possible combinations of start and goal locations (significantly exceeding typical working memory capacity); such that specific paths between the same start and goal locations were repeated in less than 20% of trials, on average (with completely novel paths taken throughout the task).

In addition, we found that theta phase modulated fast gamma amplitude in the entorhinal cortex during the encoding of new paths and slow gamma amplitude in the hippocampus during the retrieval of previously traversed paths. This replicates previous rodent (20, 36–40) and human (27, 28, 41) studies which have shown that fast and slow gamma power and phase–amplitude coupling originating from the entorhinal cortex and intrahippocampal projections are preferentially engaged during memory encoding and retrieval, respectively. Numerous rodent studies have also demonstrated how the hippocampal theta rhythm organizes sequences of place and grid cell firing in each oscillatory cycle to represent upcoming behavioral trajectories (21–24, 42, 43), and that the length of spatial trajectories encoded by these theta sweeps is greater during navigation toward distant goals (25, 26). As such, our findings are consistent with the hypothesis that theta sequences of upcoming locations in the human hippocampus are maintained by phase–amplitude coupling, with fast gamma from the entorhinal cortex and slow gamma from intrahippocampal sources dominating during the planning of new routes and retrieval of previous routes, respectively.

In summary, we have demonstrated that hippocampal theta oscillations code for distance to a hidden goal during both spatial planning and subsequent navigation. In addition, we have shown that hippocampal theta modulation of gamma amplitude in distinct slow and fast bands increases iteratively as a hidden goal is approached during navigation of novel and previously traversed paths, respectively. These findings suggest that hippocampal theta–gamma phase–amplitude coupling coordinates sequences of actions using different gamma bands for mnemonic and prospective planning of goal-directed navigation across mammalian species.

## Methods

### Participants.

Twenty-seven healthy participants were recruited to participate in this experiment. Ethical approval was granted by the local research ethics committee at University College London. All participants gave written informed consent and were compensated for taking part. Two participants were excluded due to issues with the behavioral task, one was excluded for poor performance, and one for excessive MEG signal artifact. Of the remaining 23 participants, 16 were male and 18 were right-handed, with a mean ± SD age of 24.1 ± 4.89 y (range 18 to 35 y).

### Experimental Design.

In the abstract navigation task, participants moved across a 4 × 4 grid comprised of 16 images to reach a specific goal location in each trial (Fig. 1 *A* and *B*). Participants were instructed to navigate to the goal location using the shortest possible path—moving up, down, left, or right in each step using a button box. Importantly, participants never saw the whole map, which remained fixed across the entire experiment: at any time, only the four locations that were immediately north, south, east, and west of their current location were shown on screen (except at the edge or corners of the map, where fewer locations were available). As such, participants had to learn the map layout during navigation in each trial. The correspondence between map locations and visual stimuli was randomized for each participant.

In each trial, participants were presented with a goal image for 3 s (“cue period”) after a 3 s fixation period and then placed on the map but frozen in their current location for another 3 s (“frozen period”) before being allowed to navigate freely (Fig. 1*A*). The goal location in each trial was chosen at random from all locations that were at least three steps away from the current location on the map, and the start location in each trial was always the goal location from the previous trial. In all trials apart from the first trial in each task block, therefore, participants knew the start and goal location they had to navigate between during the cue period. The goal image was also displayed in the top right-hand corner of the screen throughout navigation in each trial, changing to a green tick when the goal location was reached. Upon reaching the goal, the screen was frozen for 1 s on the goal image before the start of the next trial. Each participant completed 100 trials in the MEG scanner across three task blocks.

### Behavioral Analysis.

To evaluate participants’ performance, correct trials were defined as those in which the shortest possible path to the goal image was taken (noting that there are often multiple different shortest paths, Fig. 1*C*). Trial-by-trial performance was also characterized by path tortuosity, defined as the total number of steps taken divided by the length of the shortest possible path (equal to one for correct trials, and greater than one for incorrect trials). We define “novel” paths as those in which at least one transition had not been experienced before in either direction. Thereafter, the familiarity of each path is quantified as the number of transitions on that path that have been previously experienced divided by the total number of transitions made. Reaction times are defined as the time taken to make the first button press after the end of the frozen period.

### MEG Acquisition and Preprocessing.

MEG data were recorded at a sample rate of 600 Hz using a whole-head 275-channel axial gradiometer system (CTF-Omega, VS Med Tech), while participants sat upright in a magnetically shielded room. Head position coils were attached to the nasion, left and right preauricular sites for anatomical coregistration.

MEG data were preprocessed using SPM12 (44), Fieldtrip (45), and custom MATLAB code. First, data were imported into MATLAB and cropped to the start and end of the task period. Data were then high-pass (0.1 Hz) and notch (48 to 52 Hz) filtered to remove slow drift and mains noise, respectively. Physiological artifacts related to eye blinks and lateral eye movements were identified and removed using independent components analysis implemented in Fieldtrip and EEGLAB (46). Two bad channels were identified in recordings from all participants using a generalized extreme studentized deviate test adopted from the OHBA software library (OSL) and removed from all subsequent analyses. Finally, data from each trial were “epoched” into shorter time windows: from −5 to 8 s around the onset of each cue period (“spatial planning” epochs); and −3 to 3 s around the onset of each image during subsequent movement across the map (“navigation” epochs). Data from each participant were then merged across task blocks.

### Time-Frequency Analysis.

Estimates of dynamic changes in oscillatory power during task periods of interest were generated using SPM12. Specifically, power estimates were obtained for 20 logarithmically spaced frequency bands in the 1 to 30 Hz range by convolving the signal with a five-cycle Morlet wavelet. Power values were log-transformed and then baseline-corrected before being submitted to subsequent analyses. Time-frequency data for the 13 s “spatial planning” epochs were baseline corrected using mean power in each frequency band during a −1.5 to −0.5 s window prior to the onset of the cue period [i.e., corresponding to a 1.5 to 2.5 s window during the preceding fixation period] and then cropped to the 0 to 3 s cue period to avoid edge effects. Time-frequency data for the 6 s “navigation” epochs were baseline corrected using mean power in each frequency band during a −1 to −0.5 s window prior to each button press and then cropped to a −1 to 1 s window around each button press to avoid edge effects. Navigation epochs in which two buttons were pressed simultaneously were excluded from subsequent analysis.

### MEG Source Reconstruction.

MEG source reconstruction was conducted using the linearly constrained minimum variance (LCMV) beamformer (47) in SPM12 with a single-shell forward model to generate maps of mean source power on a 10-mm grid coregistered to MNI coordinates. As the LCMV beamformer requires a baseline period with a duration equal to the time window of interest, the 3 s fixation period prior to cue onset was used as a baseline for cue period power contrasts, while no baseline was selected for the navigation period. In some cases, results were small volume corrected using anatomical masks generated by the WFU PickAtalas (48) with hippocampal regions defined by the Automated Anatomical Labelling Atlas (49), or probabilistic masks of the entorhinal cortex from previous studies thresholded at 40% (50, 51).

### MEG Source Regression.

To identify relationships between behavior and oscillatory power in source space, we conducted linear regression between the trial-by-trial power estimates in each voxel obtained by source localization and distance to the goal using the multiple linear regression module implemented in SPM. Importantly, power estimates for both the cue and navigation periods were not baseline-corrected in this case. In addition, we included the trial number as a nuisance regressor to remove any potential effect of slow signal drift. Images of regression coefficients across voxels were then submitted to a one-sample *t* test across participants with an uncorrected threshold of *P* < 0.001, and results were reported if they passed a family-wise error (FWE) correction threshold of *P* < 0.05 at the cluster level, either across the whole brain or following small volume correction. All clusters that passed the uncorrected threshold of *P* < 0.001 at the whole brain level for each analysis are listed in *SI Appendix*, Table S1, with labels taken from the Neuromorphometrics atlas in SPM12.

### PAC Analysis.

To examine PAC between 2 to 5 Hz theta phase and the amplitude of slow and fast gamma bands (30 to 70 Hz and 70 to 140 Hz, respectively), we first reconstructed the filtered signal in each frequency band of interest in each source space voxel using weights from the LCMV beamformer and a zero phase, finite impulse response filter. Next, we extracted the analytical signal using the Hilbert transform and computed the resultant vector length of theta phase weighted by instantaneous gamma amplitude across all time bins in each voxel for each window of interest (52, 53). To remove any potential confound caused by concurrent trial-by-trial changes in oscillatory power, we constructed a general linear model to predict phase–amplitude coupling from theta and gamma power in each trial and then generated trial-by-trial images of the residuals to enter into our regression analysis against distance to the goal with trial number as a nuisance regressor. Finally, images of regression coefficients across voxels were submitted to a one-sample *t* test across participants with an uncorrected threshold of *P* < 0.001, and results were reported if they passed a FWE correction threshold of *P* < 0.05 at the cluster level, either across the whole brain or following small volume correction.

### Aperiodic Slope.

To quantify aperiodic (1/f) slope, we used Welch’s method to estimate the power spectrum of the 3 s cue period or 2 s window around button pressing during navigation in the 1 to 150 Hz range, averaged those power spectra across all trials with the same goal distance, and then applied the FOOFF algorithm (54). Finally, linear regression was used to assess whether aperiodic (1/f) slope changes consistently with goal distance for each participant, and regression coefficients across participants were submitted to a one-sample *t* test with an uncorrected threshold of *P* < 0.05.

### Event Related Fields.

To quantify theta band amplitude of the evoked response, we used the same approach as in our other analyses but averaged the MEG signal across all trials with the same goal distance before generating time-frequency representations. We then used linear regression to assess whether 2 to 5 Hz or 6 to 9 Hz theta band amplitude of the evoked response covaried with goal distance during each period of interest, and one-sample *t* tests across participants to ask whether the regression coefficients differed significantly from zero. Importantly, to match trial numbers when averaging the MEG signal across trials, we restricted these analyses to goal distances of four steps or less (as there were relatively few trials with five step paths, see Fig. 1*D*) and subsampled trials to be consistent across goal distances. The subsampling process was repeated 100 times and resultant theta power estimates averaged across all iterations before being entered into linear regression for each participant.

### Asymmetric Index.

To quantify theta wave asymmetry, we filtered the signal from each epoch in the 2 to 5 Hz band using a zero-phase Butterworth filter, then identified local minima and maxima of the filtered signal in the 3 s cue period or 2 s window centered on each button press. Those timestamps were used to compute the asymmetry index for each trial (55, 56), which is defined as the difference in the duration of the ascending and descending phases of the cycle (i.e., trough to peak and peak to trough, respectively), divided by their sum. We then used linear regression to assess whether theta wave asymmetry covaried with goal distance during each period of interest (with trial number included as a nuisance regressor, as in the corresponding phase–amplitude coupling analysis), and one-sample *t* tests across participants to ask whether the regression coefficients differed significantly from zero.

## Supplementary Material

Appendix 01 (PDF)

## Data Availability

Data and code are available in the University College London Research Data Repository (57).
