# Supplementary material for: Human hippocampal theta–gamma coupling coordinates sequential planning during navigation
Source: Proc Natl Acad Sci U S A. 2026 Feb 27;123(9):e2513547123. doi: 10.1073/pnas.2513547123 (PMC12956831; doi:10.1073/pnas.2513547123)
Supplement: Supplementary file 1 — Appendix 01 (PDF) [file pnas.2513547123.sapp.pdf]

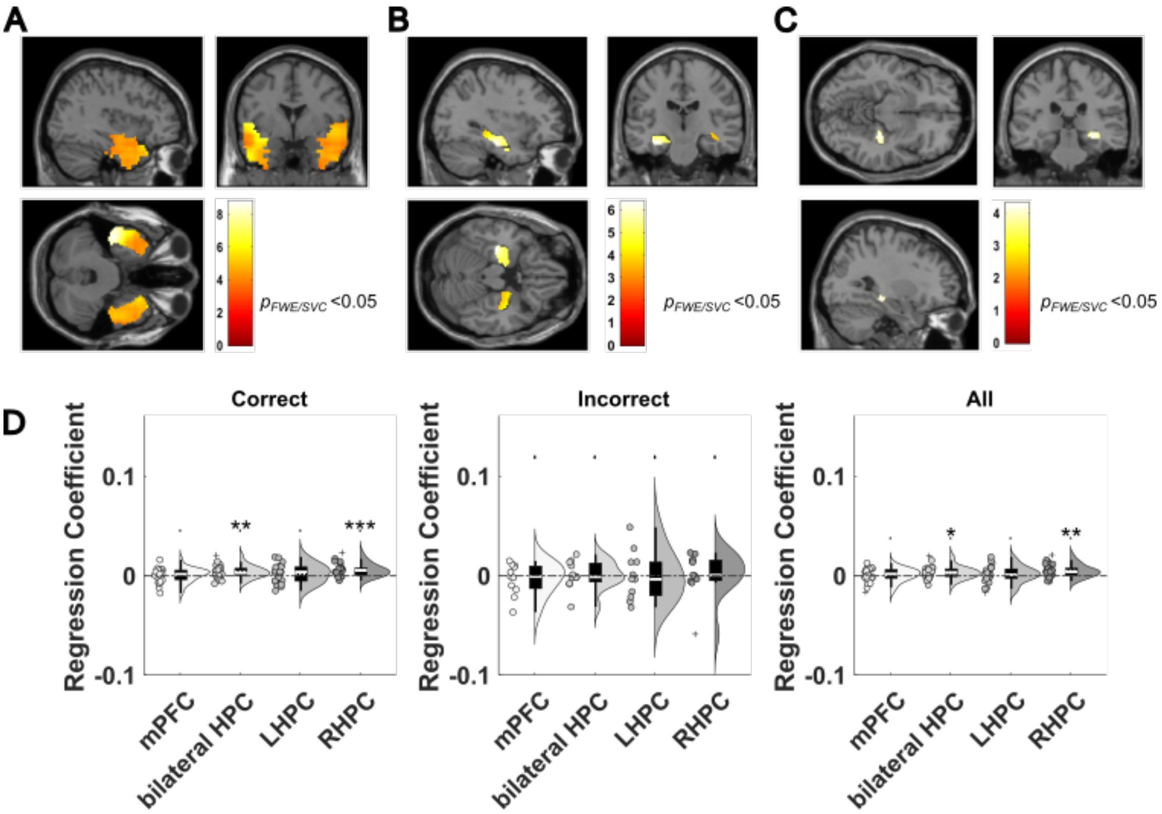

Figure S1. Frontotemporal theta power increases during spatial planning. **(A)** Source localisation of increase in 2-5 Hz theta power during the 3s cue period, masked and small volume corrected with a bilateral temporal lobe mask (visualised at $p_{uncorr} < 0.001$). **(B)** Source localisation of increase in 2-5Hz theta power during the 3s cue period, masked and small volume corrected with a bilateral hippocampal mask (visualised at $p_{uncorr} < 0.001$). **(C)** Source localisation of the relationship between 2-5 Hz theta power during the 3s cue period and shortest path distance to the goal for all correct trials, masked and small volume corrected with a bilateral hippocampal mask (visualised at $p_{uncorr} < 0.001$). **(D)** Regression coefficients between 2-5Hz theta power during planning and shortest path distance to the goal extracted from pre-defined anatomical ROIs across correct trials (left panel), incorrect trials (middle panel), and all trials (right panel). Data shown for the medial prefrontal cortex (mPFC, all $p > 0.5$), bilateral hippocampus (correct trials: $t(22) = 2.89$, $p = 0.0085$, one-sample t-test, Cohen's d = 0.581; incorrect trials: $t(22) = 0.186$, $p = 0.856$, one-sample t-test, Cohen's d = 0.0517; all trials: $t(22) = 2.33$, $p = 0.0291$, one-sample t-test, Cohen's d = 0.47), right hippocampus (RHPC, correct trials: $t(22) = 4.11$, $p < 0.001$, one-sample t-test, Cohen's d = 0.827; incorrect trials: $t(22) = 0.20$, $p = 0.843$, one-sample t-test, Cohen's d = 0.0565; all trials: $t(22) = 3.57$, $p = 0.0017$, one-sample t-test, Cohen's d = 0.719) and left hippocampus (LHPC, all $p > 0.31$). No significant difference is observed between correct trials and incorrect trials in any ROIs (all $p > 0.36$). The colour bar in panels **A, B,** and **C** shows t-statistics. Box plots show mean (dashed) and median (solid line), lower and upper quartiles (top and bottom of box), minimum and maximum values (excluding outliers, top and bottom whiskers) across participants unless otherwise stated. * = p <0.05, ** = p <0.01, *** = p <0.001.

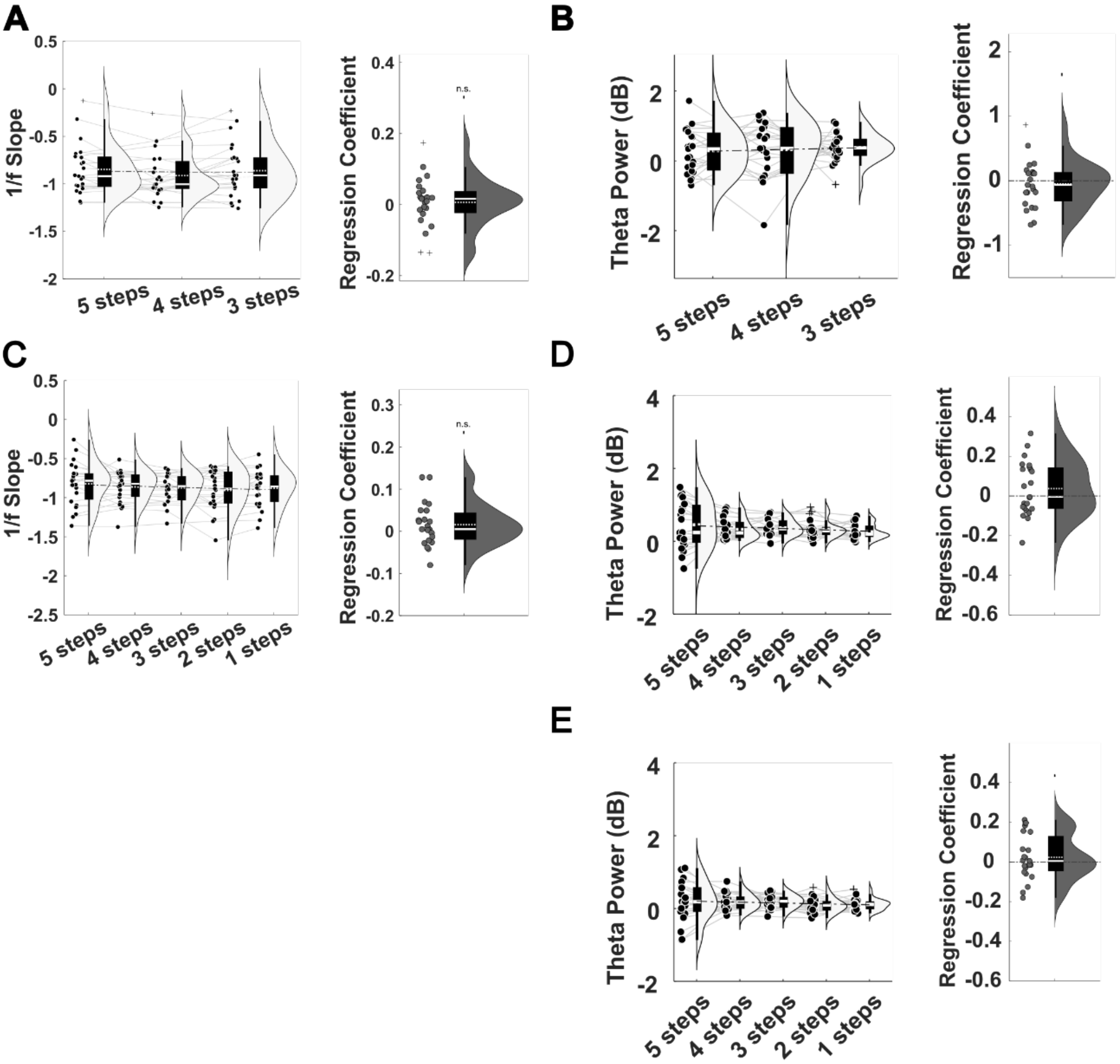

Figure S2. Relationship between aperiodic slope of the power spectrum / theta band amplitude of the ERF and distance to the goal during planning and navigation. **(A)** There is no consistent relationship between the aperiodic slope of the power spectrum and shortest path length to the goal during the planning period of correct trials (t(22) = 0.421, *p* = 0.677, one-sample t-test, Cohen's d = 0.0849). Data shown for the MEG sensor that exhibited the strongest relationship between theta power and shortest path length to the goal during the same period (MRT16). **(B)** Theta band (2-5Hz) amplitude of the ERF across participants for each shortest path length to the goal during the planning period of correct trials for the same MEG sensor (MRT16), which shows no consistent relationship across participants (t(22) = -0.627, *p* = 0.537, one-sample t-test, Cohen's d = 0.126). **(C)** There is no consistent relationship between the aperiodic slope of the power spectrum and distance remaining to the goal during navigation in correct trials (t(22) = 1.51, p = 0.145, one-sample t-test, Cohen's d = 0.303). Data shown for the MEG sensor that exhibited the strongest relationship between theta power and shortest path length to the goal during the same period (MRT57). **(D)** Theta band (2-5Hz) amplitude of the ERF across participants for each

distance remaining to the goal during navigation in correct trials for the same MEG sensor, which shows no consistent relationship across participants ($t(22) = 1.33$, $p = 0.196$, one-sample t-test; Cohen's d = 0.268). **(E)** Theta band (6-9Hz) amplitude of the ERF across participants for each distance remaining to the goal during navigation in correct trials for the same MEG sensor, which shows no consistent relationship across participants ($t(22) = 1.00$, $p = 0.329$, one-sample t-test; Cohen's d = 0.201). Box plots show mean (dashed) and median (solid line), lower and upper quartiles (top and bottom of box), minimum and maximum values (excluding outliers, top and bottom whiskers) across participants unless otherwise stated.

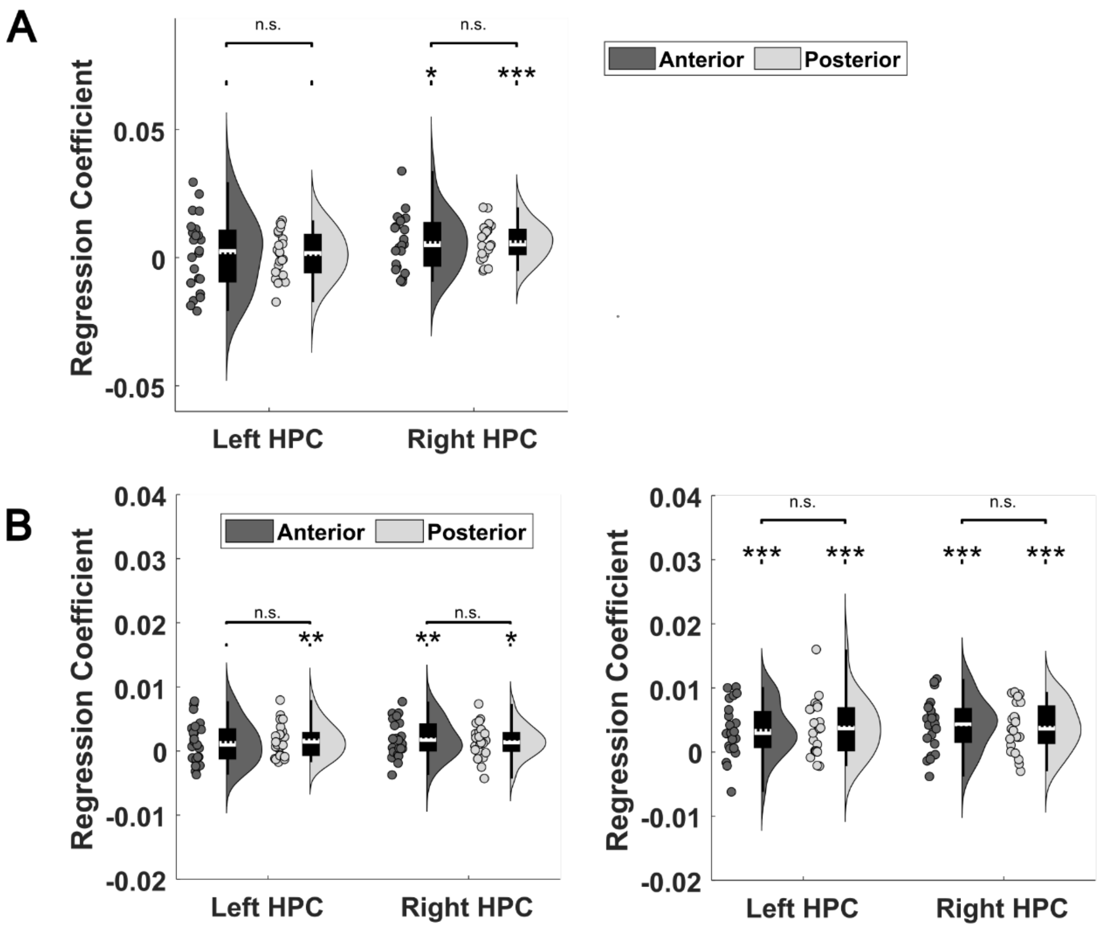

Figure S3. Theta power in both the anterior and posterior hippocampus covaries with distance to the goal during planning and navigation. **(A)** Regression coefficients for average 2-5Hz theta power extracted from anatomically defined left and right, anterior and posterior hippocampal masks against distance to the goal during the cue period, for correct trials only. No difference in the strength of the theta power code for goal distance is seen in either case (both $p > 0.74$). **(B)** Regression coefficients for average 2-5Hz (left panel) and 6-9Hz (right panel) theta power extracted from anatomically defined left and right, anterior and posterior hippocampal masks against distance to the goal during navigation, for correct trials only. No difference in the strength of the theta power code for goal distance is seen in either case (all $p > 0.18$). Box plots show mean (dashed) and median (solid line), lower and upper quartiles (top and bottom of box), minimum and maximum values (excluding outliers, top and bottom whiskers) across participants unless otherwise stated. * = $p < 0.05$, ** = $p < 0.01$, *** = $p < 0.001$.

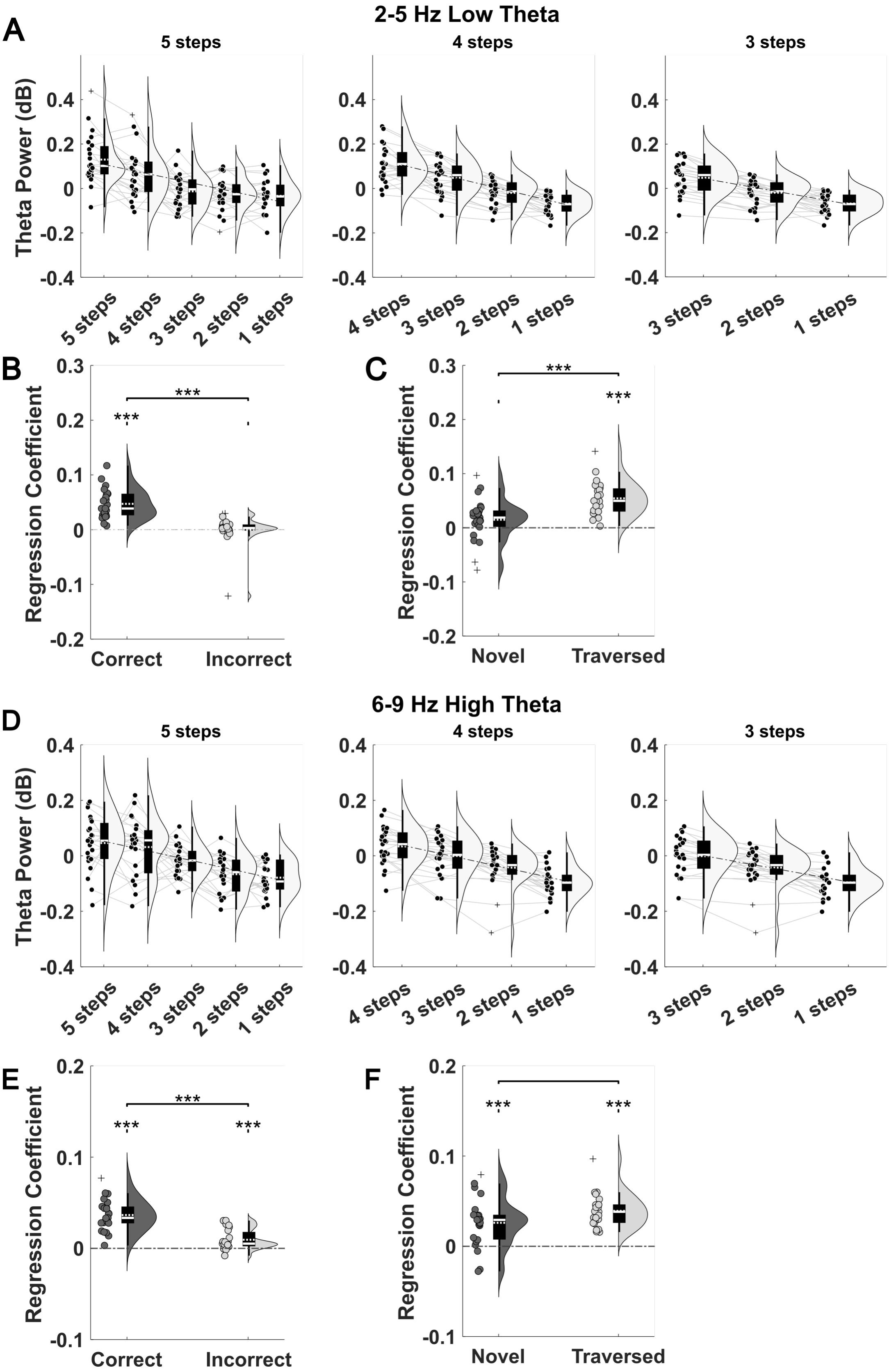

2-5 Hz Low Theta
A
5 steps
4 steps
3 steps
Theta Power (dB)
0.4
0.2
0
-0.2
-0.4
5 steps
4 steps
3 steps
2 steps
1 steps
B
C
Regression Coefficient
0.3
0.2
0.1
0
-0.1
-0.2
***
Correct
Incorrect
Novel
Traversed
6-9 Hz High Theta
D
E
F

Figure S4. Scalp level theta power covaries with distance to the goal during navigation. **(A)** 2-5 Hz theta power averaged across all sensors for all correct trials, split by the remaining distance to the goal in descending order during navigation. Left panel: correct five-step paths; middle panel: correct four-step paths; right panel: correct three-step paths. **(B)** Regression coefficient for average 2-5 Hz theta power against distance to the goal for correct (t(22) = 8.28, $p < 0.001$, one-sample t-test, Cohen's d = 1.67) and incorrect trials (t(22) = 0.0098, $p = 0.99$, one-sample t-test, Cohen's d = 0.002). This effect is significantly stronger in correct trials (t(22) = 6.22, $p < 0.001$, paired-t-test, Cohen's d = 1.64). **(C)** Regression coefficient for average 2-5Hz theta power against distance to the goal for correct trials that use novel (t(22) = 1.82, $p = 0.0827$, one-sample t-test, Cohen's d = 0.37) or previously traversed paths (t(22) = 8.33, $p < 0.001$, one-sample t-test, Cohen's d = 1.68). This effect is significantly stronger for previously traversed paths (t(22) = 4.39, $p < 0.001$, paired t-test, Cohen's d = 1.07). **(D)** 6-9 Hz theta power averaged across all sensors for all correct trials, split by the remaining distance to the goal in descending order during navigation. Left panel: correct five-step paths; middle panel: correct four-step paths; right panel: correct three-step paths. **(E)** Regression coefficient for average 6-9 Hz theta power against distance to the goal for correct (t(22) = 10.2, $p < 0.001$, one-sample t-test, Cohen's d = 2.05) and incorrect trials (t(22) = 4.16, $p < 0.001$, one-sample t-test, Cohen's d = 0.84). This effect is significantly stronger in correct trials (t(22) = 7.90, $p < 0.001$, paired t-test, Cohen's d = 1.82). **(F)** Regression coefficient for average 6-9 Hz theta power against distance to the goal for correct trials that use novel (t(22) = 4.63, $p < 0.001$, one-sample t-test, Cohen's d = 0.93) or previously traversed paths (t(22) = 10.2, $p < 0.001$, one-sample t-test, Cohen's d = 2.05). The effect is marginally stronger for previously traversed paths (t(22) = 2.06, $p = 0.0512$, paired t-test, Cohen's d = 0.55). Box plots show mean (dashed) and median (solid line), lower and upper quartiles (top and bottom of box), minimum and maximum values (excluding outliers, top and bottom whiskers) across participants unless otherwise stated. *** = p <0.001.

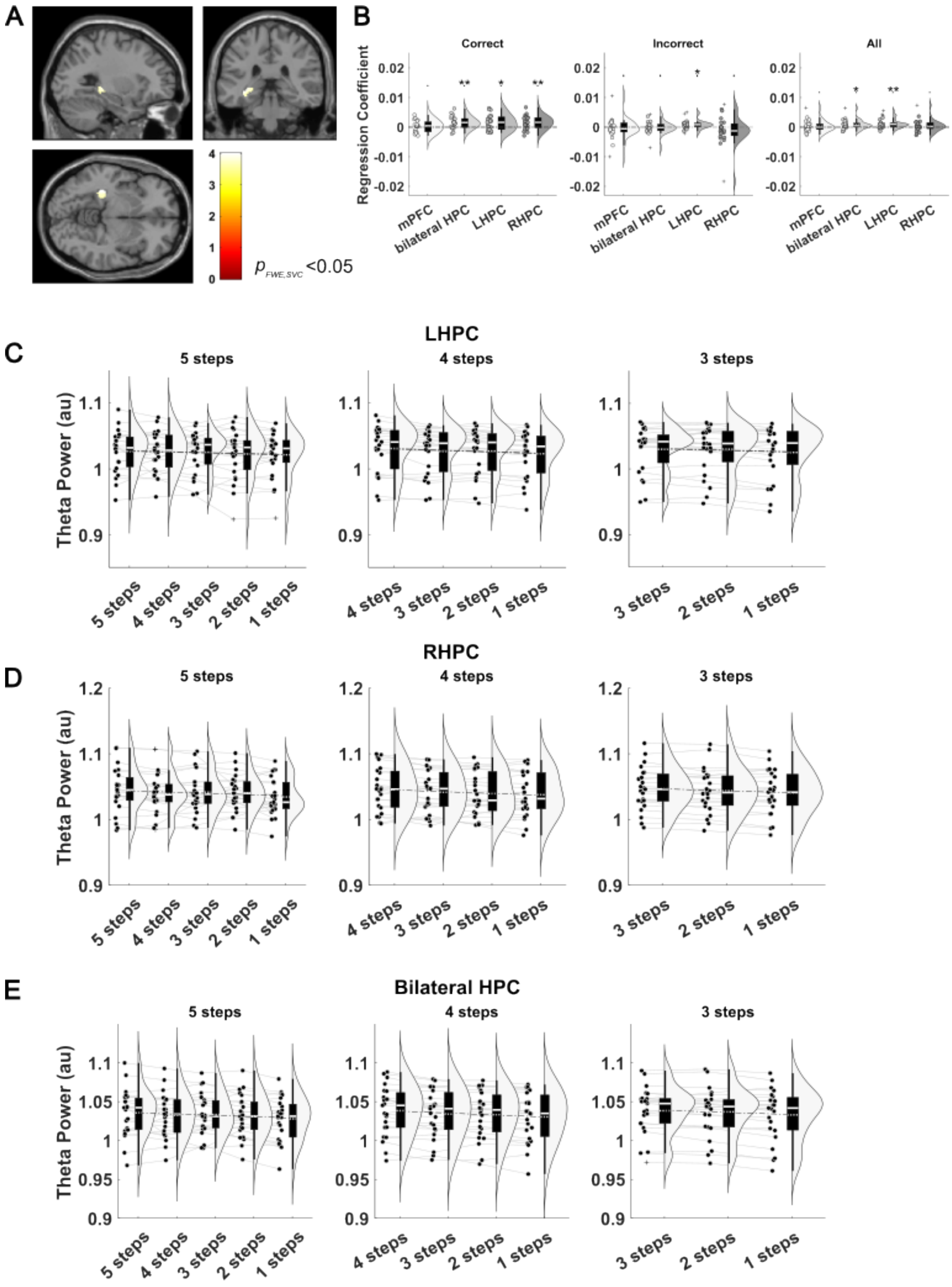

Figure S5. Source level 2-5Hz theta power covaries with distance to the goal during navigation. **(A)** Source localisation of the relationship between 2-5 Hz theta power and shortest path distance to the goal for all correct trials within a bilateral HPC mask (visualised at $p_{uncorr} < 0.001$), with a peak in the left hippocampus ([-34, -34, -2];

Z = 3.43, $p_{FWE, SVC} < 0.05$). **(B)** Regression coefficients between 2-5Hz theta power during navigation and shortest path distance to the goal extracted from pre-defined anatomical ROIs across correct trials (left panel), incorrect trials (middle panel), and all trials (right panel). Data shown for the medial prefrontal cortex (mPFC, all $p > 0.44$), bilateral hippocampus (correct trials: t(22) = 3.51, $p = 0.002$, one-sample t-test, Cohen's d = 0.707; incorrect trials: t(22) = -0.20, $p = 0.844$, one-sample t-test, Cohen's d = 0.0401; all trials: t(22) = 2.43, $p = 0.0236$, one-sample t-test, Cohen's d = 0.490), left hippocampus (LHPC, correct trials: t(22) = 2.76, $p = 0.0114$, one-sample t-test, Cohen's d = 0.556; incorrect trials: t(22) = 2.63, $p = 0.0152$, one-sample t-test, Cohen's d = 0.530; all trials: t(22) = 3.49, $p = 0.0021$, one-sample t-test, Cohen's d = 0.702) and right hippocampus (RHPC, correct trials: 3.26, $p = 0.0036$, one-sample t-test, Cohen's d = 0.657; incorrect trials: t(22) = -0.980, $p = 0.338$, one-sample t-test, Cohen's d = 0.20; all trials: t(22) = 1.12, $p = 0.273$, one-sample t-test, Cohen's d = 0.226). Distance to goal coding is significantly stronger in correct versus incorrect trials in the bilateral hippocampus (t(22) = 3.37, $p = 0.0028$, paired t-test, Cohen's d = 0.730) and right hippocampus (t(22) = 2.67, $p = 0.014$, paired t-test, Cohen's d = 0.635), while there is no significant difference in mPFC or left hippocampus (both $p > 0.29$). **(C, D, E)** 2-5 Hz theta power extracted from (C) left hippocampus, (D) right hippocampus, and (E) bilateral hippocampus split by remaining distance to the goal in descending order during navigation. Left panel: correct five-step paths; middle panel: correct four-step paths; right panel: correct three-step paths. The colour bar in panel **A** shows t-statistics. Box plots show mean (dashed) and median (solid line), lower and upper quartiles (top and bottom of box), minimum and maximum values (excluding outliers, top and bottom whiskers) across participants unless otherwise stated. * = p <0.05, ** = p <0.01.

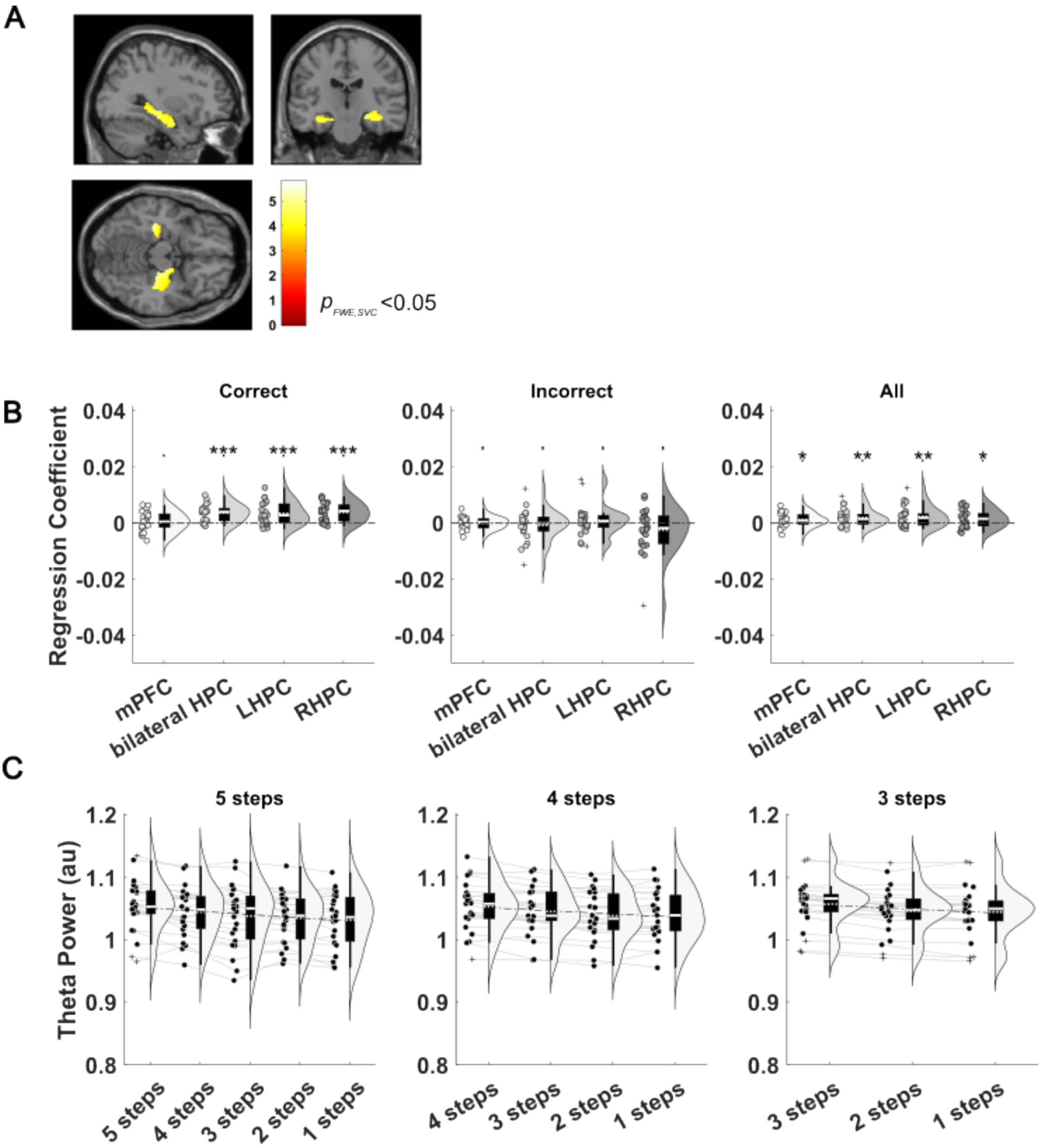

Figure S6: Source level 6-9Hz theta power covaries with distance to the goal during navigation. **(A)** Source localisation of the relationship between 6-9 Hz theta power and shortest path distance to the goal for all correct trials within a bilateral HPC mask (visualised at $p_{uncorr} < 0.001$), with a global peak in the right hippocampus (16, -10, -18; Z = 4.47, $p_{FWE,\ SVC} < 0.05$). **(B)** Regression coefficients between 6-9Hz theta power during navigation and shortest path distance to the goal extracted from pre-defined anatomical ROIs across correct trials (left panel), incorrect trials (middle panel), and all trials (right panel). Data shown for the medial prefrontal cortex (mPFC, correct and incorrect trials both $p > 0.53$; all trials: t(22) = 2.16, p = 0.0416, one-sample t-test, Cohen's d = 0.436), bilateral hippocampus (correct trials: t(22) = 5.709, $p < 0.001$, one-sample t-test, Cohen's d = 1.15; incorrect trials: -0.724, p = 0.477, one-sample t-test, Cohen's d = 0.146; all trials: t(22) = 3.02, p = 0.0063, one-

sample t-test, Cohen's d = 0.608), left hippocampus (LHPC, correct trials: $t(22) = 4.10$, $p < 0.001$, one-sample t-test, Cohen's d = 0.826; incorrect trials: $t(22) = 0.693$, $p = 0.50$, one-sample t-test, Cohen's d = 0.140; all trials: $t(22) = 3.00$, $p = 0.0066$, one-sample t-test, Cohen's d = 0.604) and right hippocampus (RHPC, correct trials: $t(22) = 5.52$, $p < 0.001$, one-sample t-test, Cohen's d = 1.11; incorrect trials: $t(22) = -1.45$, $p = 0.161$, one-sample t-test, Cohen's d = 0.292; all trials: $t(22) = 2.28$, $p = 0.0325$, one-sample t-test, Cohen's d = 0.460). Distance to goal coding is significantly stronger in correct versus incorrect trials in the bilateral hippocampus ($t(22) = 4.10$, $p < 0.001$, paired t-test, Cohen's d = 0.949), right ($t(22) = 3.53$, $p = 0.0029$, paired t-test, Cohen's d = 0.969) and left hippocampus ($t(22) = 2.60$, $p = 0.0164$, paired t-test, Cohen's d = 0.512), while there is no significant difference in mPFC ($p > 0.56$, paired t-test). **(C)** 6-9 Hz theta power extracted from the bilateral hippocampus split by the remaining distance to the goal in descending order during navigation. Left panel: correct five-step paths; middle panel: correct four-step paths; right panel: correct three-step paths. The colour bar in panel **A** shows t-statistics. Box plots show mean (dashed) and median (solid line), lower and upper quartiles (top and bottom of box), minimum and maximum values (excluding outliers, top and bottom whiskers) across participants unless otherwise stated. * = $p < 0.05$, ** = $p < 0.01$, *** = $p < 0.001$.

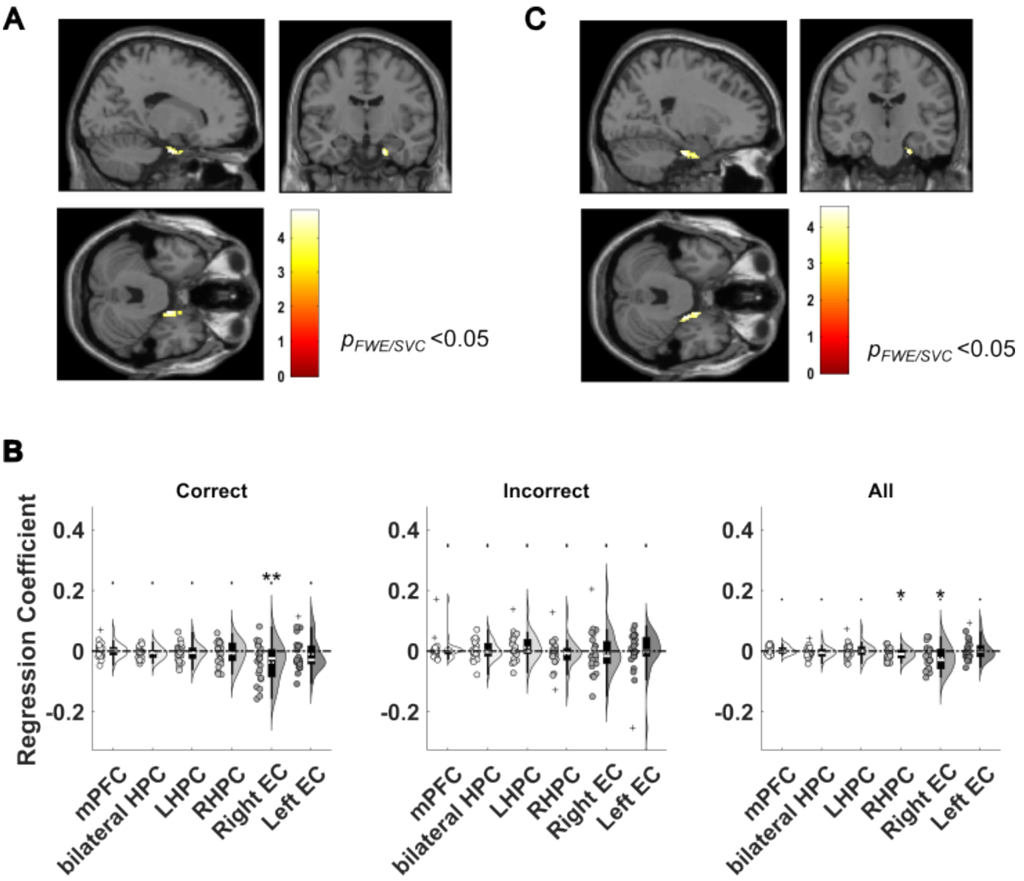

Figure S7. Low theta–fast gamma phase-amplitude coupling (PAC) covaries with distance to the goal during navigation. **(A)** Source localisation of the relationship between 2-5Hz theta - 70-140 Hz gamma PAC and distance to the goal for all correct trials (visualised at $p_{uncorr} < 0.001$) within a pre-defined right entorhinal mask. **(B)** Regression coefficients between 2-5Hz theta - 70-140 Hz gamma PAC during navigation and distance to the goal extracted from pre-defined anatomical ROIs across correct trials (left panel), incorrect trials (middle panel), and all trials (right panel). Data shown for the medial prefrontal cortex (mPFC, all $p > 0.38$), bilateral hippocampus (all $p > 0.09$), right hippocampus (RHPC, correct and incorrect trials separately both $p > 0.32$, all trials: $t(22) = -2.46$, $p = 0.0224$, one-sample t-test, Cohen's d = 0.494), left hippocampus (LHPC, all $p > 0.21$), right entorhinal cortex (Right EC, correct trials: $t(22) = -2.91$, $p = 0.0082$, one-sample t-test, Cohen's d = 0.585; incorrect trials: $t(22) = -0.340$, $p = 0.737$, one-sample t-test, Cohen's d = 0.068; all trials: $t(22) = -2.74$, $p = 0.0119$,one-sample t-test, Cohen's d = 0.552) and left entorhinal cortex (Left EC, all $p > 0.37$). There is no significant difference between correct trials and incorrect trials in any of the ROIs (all $p > 0.18$).**(C)** Source localisation of the relationship between 2-5Hz theta - 70-140 Hz gamma PAC and distance to the goal for all correct and novel trials (visualised at $p_{uncorr} < 0.001$) within a pre-defined right entorhinal mask. The colour bar in panels **A** and **B** show t-statistics. Box plots show mean (dashed) and median (solid line), lower and upper quartiles (top and bottom of box), minimum and maximum values (excluding outliers, top and bottom whiskers) across participants unless otherwise stated. * = $p < 0.05$, ** = $p < 0.01$.

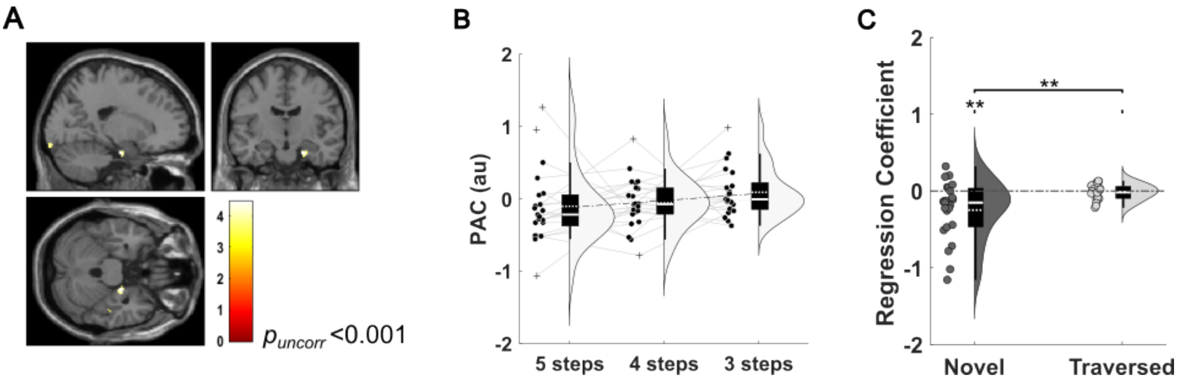

Figure S8. Theta-gamma phase-amplitude coupling covaries with distance to the goal during planning. **(A)** Source localisation of the 2-5Hz theta - 70-140 Hz fast gamma phase-amplitude coupling vs goal distance relationship for all correct trials (visualised at $p_{uncorr} < 0.001$). A significant cluster is observed in the right anterior temporal lobe ([20, -12, -26], Z = 3.72, $p_{uncorr} < 0.001$). **(B)** Average theta-fast gamma PAC extracted from a pre-defined right entorhinal mask for all correct trials, split by distance to the goal in descending order during planning. **(C)** Regression coefficients between theta-fast gamma PAC and goal distance in an anatomically defined right entorhinal mask, for all correct trials preceding novel (t(22) = -3.10, p = 0.0052, one-sample t-test, Cohen's d = 0.624) and previously traversed (t(22) = -0.806, p = 0.429, one-sample t-test, Cohen's d = 0.162) paths. The PAC vs distance to goal relationship is significantly stronger in trials preceding novel vs previously traversed paths (t(22) = -2.90, p = 0.0082, paired t-test, Cohen's d = 0.795). The colour bar in panel **A** shows t-statistics. Box plots show mean (dashed) and median (solid line), lower and upper quartiles (top and bottom of box), minimum and maximum values (excluding outliers, top and bottom whiskers) across participants unless otherwise stated. ** = p <0.01.

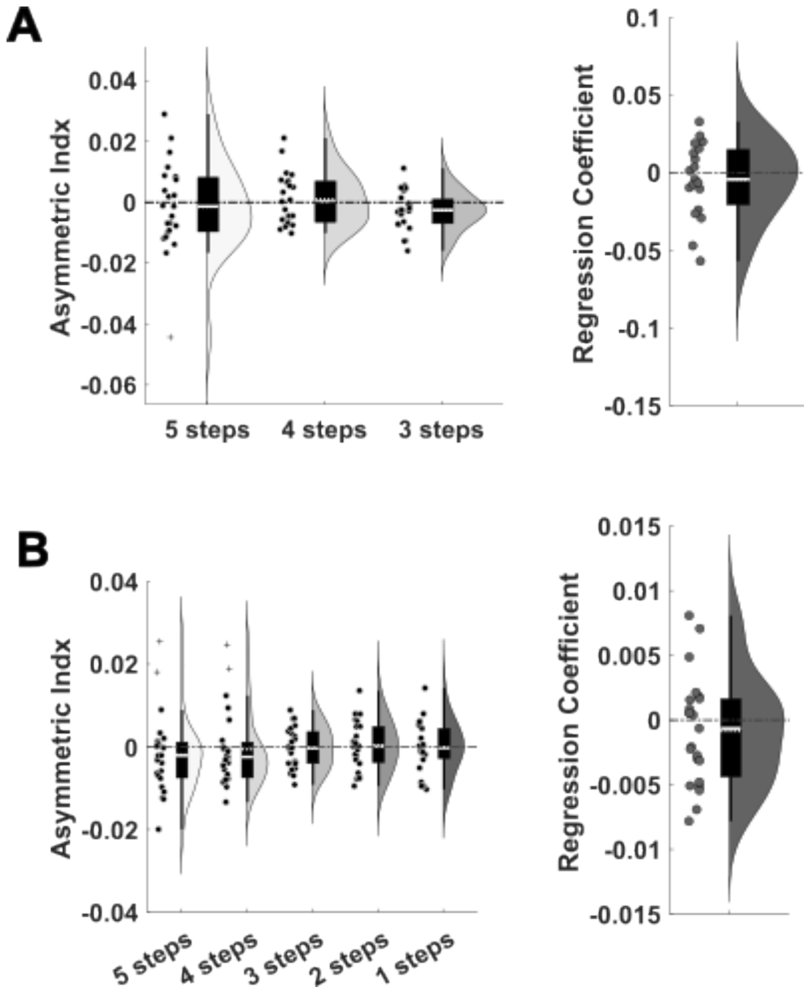

Figure S9. No relationship between theta wave asymmetry and distance to the goal. **(A)** Mean theta wave asymmetry index across participants for each distance remaining to the goal during the planning period of correct trials, using data from the MEG sensor with the strongest relationship between theta-gamma phase-amplitude coupling and goal distance during this period (MRT25). This shows no consistent relationship between theta wave asymmetry and distance to the goal across participants (t(18) = -0.804, *p* = 0.432, one-sample t-test; Cohen's d = 0.177). **(B)** Mean theta wave asymmetry index across participants for each distance remaining to the goal during navigation in correct trials, using data from the MEG sensor with the strongest relationship between theta-gamma phase-amplitude coupling and goal distance during navigation (MRC16). This shows no consistent relationship between theta wave asymmetry and distance to the goal across participants (t(22) = -0.964, *p* = 0.345, one-sample t-test, Cohen's d = 0.194). Box plots show mean (dashed) and median (solid line), lower and upper quartiles (top and bottom of box), minimum and maximum values (excluding outliers, top and bottom whiskers) across participants unless otherwise stated.

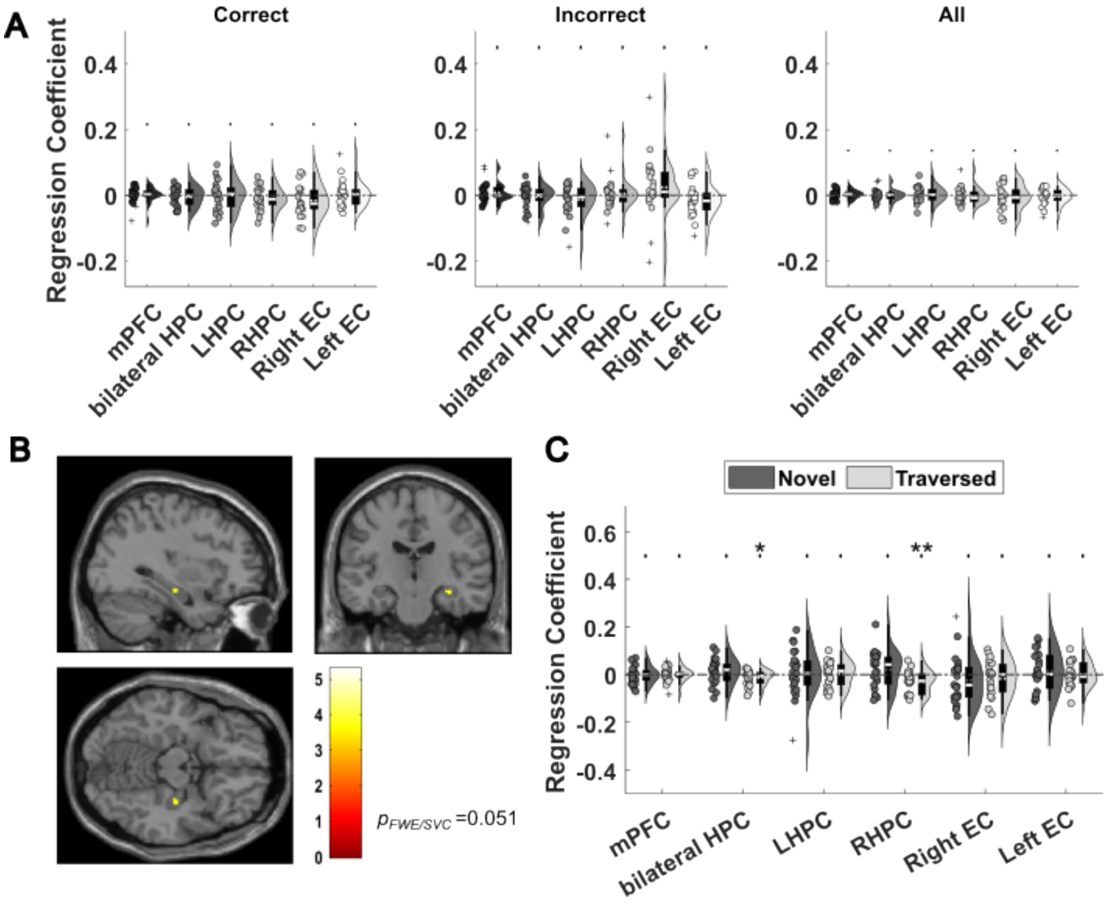

Figure S10. Low theta - slow gamma phase-amplitude coupling covaries with distance to goal during navigation along previously traversed paths. **(A)** Regression coefficients between 2-5Hz theta - 30-70 Hz gamma PAC during navigation and distance to the goal extracted from pre-defined anatomical ROIs across correct trials (left panel), incorrect trials (middle panel), and all trials (right panel). Data shown for the medial prefrontal cortex (mPFC), bilateral hippocampus, right hippocampus (RHPC), left hippocampus (LHPC), right entorhinal cortex (Right EC) and left entorhinal cortex (Left EC). No significant relationship is observed in any ROIs (all $p > 0.23$). **(B)** Source localisation of the relationship between 2-5Hz theta - 30-70 Hz slow gamma PAC and distance to the goal for all correct trials that used previously traversed paths (visualised at $p_{uncorr} < 0.001$) within a pre-defined right hippocampal mask. **(C)** Regression coefficient for 2-5Hz theta - 30-70 Hz slow gamma PAC against distance to the goal for correct trials that use novel or previously traversed paths in pre-defined anatomical ROIs. This effect is significantly stronger for previously traversed paths in the right hippocampus ($t(22) = -2.26$, $p = 0.0342$, paired t-test, Cohen's d = 0.751) and the bilateral hippocampus ($t(22) = -2.08$, $p = 0.050$, paired t-test, Cohen's d = 0.603), but not in any other ROIs (all $p > 0.56$). The colour bar in panel **B** shows t-statistics. Box plots show mean (dashed) and median (solid line), lower and upper quartiles (top and bottom of box), minimum and maximum values (excluding outliers, top and bottom whiskers) across participants unless otherwise stated. * = $p < 0.05$, ** = $p < 0.01$.

Cluster-level				Peak-level					x	y	z	Brain region(s)
$p_{FWE-corr}$	$p_{FDR-corr}$	K_E	P_{unc}	$p_{FWE-corr}$	$p_{FDR-corr}$	T	Z_E	p_{unc}				
<0.001	<0.001	67257	<0.001	<0.001	0.002	11.99	6.60	<0.001	-4	24	-8	Left anterior cingulate gyrus
				<0.001	0.002	11.64	6.52	<0.001	-24	-6	14	
				<0.001	0.003	10.97	6.35	<0.001	-32	-4	14	
0.005	0.176	344	0.088	0.022	0.483	5.54	4.34	<0.001	46	-18	-44	
				0.023	0.502	5.51	4.32	<0.001	50	-14	-38	Right inferior temporal gyrus
				0.024	0.506	5.50	4.32	<0.001	42	-26	-50	
0.046	0.952	2	0.924	0.045	0.909	5.15	4.13	<0.001	60	-30	6	Right cerebral white matter
0.048	0.952	1	0.952	0.046	0.924	5.14	4.12	<0.001	12	-2	8	Right cerebral white matter

Figure 2B

Cluster-level				Peak-level					x	y	z	Brain region(s)
$p_{FWE-corr}$	$p_{FDR-corr}$	K_E	P_{unc}	$p_{FWE-corr}$	$p_{FDR-corr}$	T	Z_E	p_{unc}				
0.466	0.913	121	0.466	0.314	0.928	4.24	3.59	<0.001	30	-28	-10	Right hippocampus
0.240	0.913	364	0.203	0.407	0.928	4.06	3.47	<0.001	40	-76	-4	Right cerebral white matter
				0.532	0.928	3.84	3.33	<0.001	28	-64	16	
				0.603	0.928	3.73	3.25	0.001	34	-70	2	
0.672	0.913	15	0.827	0.426	0.928	4.02	3.45	<0.001	6	-34	6	Right cerebral white matter
0.652	0.913	22	0.782	0.451	0.928	3.98	3.42	<0.001	4	-24	0	Right cerebral white matter
0.708	0.913	5	0.913	0.620	0.928	3.70	3.23	0.001	-6	-30	14	Left thalamus proper
0.672	0.913	15	0.827	0.693	0.928	3.59	3.15	0.001	40	-74	-14	Right cerebral white matter
0.729	1.000	1	0.969	0.735	1.000	3.51	3.10	0.001	16	-26	-6	Right cerebral white matter

Figure 2C

Cluster-level				Peak-level					x	y	z	Brain region(s)
$p_{FWE-corr}$	$p_{FDR-corr}$	K_E	P_{unc}	$p_{FWE-corr}$	$p_{FDR-corr}$	T	Z_E	p_{unc}				
0.015	0.531	91	0.531	0.006	0.223	4.24	3.59	<0.001	30	-28	-10	Right hippocampus

SI Appendix, Figure S1C

Cluster-level				Peak-level					x	y	z	Brain region(s)
$p_{FWE-corr}$	$p_{FDR-corr}$	K_E	P_{unc}	$p_{FWE-corr}$	$p_{FDR-corr}$	T	Z_E	p_{unc}				
0.012	0.048	2264	0.012	0.017	0.317	5.76	4.45	<0.001	52	-38	4	Right superior temporal gyrus
				0.019	0.679	5.71	4.42	<0.001	66	-24	-2	
				0.090	0.679	4.85	3.96	<0.001	68	-16	-4	
0.027	0.183	1686	0.027	0.168	0.679	4.49	3.74	<0.001	-46	-26	0	Left cerebral white matter
				0.172	0.679	4.47	3.73	<0.001	-54	-24	0	
				0.215	0.679	4.34	3.65	<0.001	-52	-34	0	
0.244	0.730	365	0.270	0.315	0.679	4.09	3.49	<0.001	60	-6	-30	Right middle temporal gyrus
				0.338	0.679	4.04	3.46	<0.001	64	-14	-26	
				0.440	0.679	3.85	3.33	<0.001	62	-24	-28	
0.533	1.000	42	0.734	0.598	0.888	3.58	3.15	0.001	44	-16	-26	Right inferior temporal gyrus
				0.602	0.888	3.58	3.14	0.001	36	-14	-24	

Figure 3C

Cluster-level				Peak-level					x	y	z	Brain region(s)
$p_{FWE-corr}$	$p_{FDR-corr}$	K_E	P_{unc}	$p_{FWE-corr}$	$p_{FDR-corr}$	T	Z_E	p_{unc}				
0.026	0.888	111	0.553	0.017	0.552	4.01	3.43	<0.001	-34	-34	-2	Left cerebral white matter
				0.018	0.552	4.00	3.43	<0.001	-36	-32	-6	
0.042	0.888	10	0.888	0.041	0.866	3.58	3.14	0.001	36	-14	-24	Right cerebral white matter

SI Appendix, Figure S5A

Cluster-level				Peak-level					x	y	z	Brain region(s)
$p_{FWE-corr}$	$p_{FDR-corr}$	K_E	P_{unc}	$p_{FWE-corr}$	$p_{FDR-corr}$	T	Z_E	p_{unc}				
<0.001	0.518	6302	<0.001	0.005	0.083	6.43	4.77	<0.001	-30	-24	-26	Left para-hippocampal gyrus
				0.009	0.090	6.06	4.60	<0.001	-32	-34	-24	
				0.019	0.140	5.67	4.40	<0.001	-46	-32	-6	
<0.001	0.518	15968	<0.001	0.005	0.083	6.42	4.77	<0.001	54	-50	-16	Right inferior temporal gyrus
				0.005	0.083	6.42	4.77	<0.001	34	-48	-20	
				0.006	0.083	6.33	4.73	<0.001	16	-8	-26	
0.246	0.544	362	0.284	0.013	0.108	5.86	4.50	<0.001	20	-84	34	Right cerebral white matter
0.305	0.869	258	0.366	0.072	0.196	4.95	4.01	<0.001	40	-60	50	Right angular gyrus
0.357	0.869	187	0.444	0.272	0.478	4.16	3.54	<0.001	58	-48	44	Right angular gyrus
				0.419	0.703	3.86	3.34	<0.001	60	-58	34	Right angular gyrus
0.551	0.975	26	0.804	0.328	0.573	4.04	3.46	<0.001	0	-2	80	Right angular gyrus
0.597	0.975	7	0.913	0.516	0.815	3.69	3.22	0.001	-34	-88	32	Right angular gyrus
0.621	0.975	1	0.975	0.620	0.991	3.52	3.10	0.001	8	8	-20	Right occipital fusiform gyrus
0.621	0.975	1	0.975	0.624	0.991	3.52	3.10	0.001	44	-68	-20	Right occipital fusiform gyrus

Figure 3F

Cluster-level				Peak-level					x	y	z	Brain region(s)
$p_{FWE-corr}$	$p_{FDR-corr}$	K_E	P_{unc}	$p_{FWE-corr}$	$p_{FDR-corr}$	T	Z_E	p_{unc}				
0.005	0.233	807	0.116	<0.001	0.138	5.81	4.47	<0.001	16	-10	-18	Right hippocampus
0.012	0.259	401	0.259	0.001	0.138	5.19	4.15	<0.001	-34	-26	-16	Left cerebral white matter

SI Appendix, Figure S6A

Cluster-level				Peak-level					x	y	z	Brain region(s)
p FWE-corr	p FDR-corr	K_E	P unc	p FWE-corr	p FDR-corr	T	Z_E	p unc				
0.255	0.201	162	0.040	0.119	0.310	5.77	4.46	<0.001	-36	-26	14	Left transverse temporal gyrus
0.677	0.420	72	0.154	0.456	0.650	4.93	4.00	<0.001	-44	-12	38	Left precentral gyrus
				0.989	0.899	3.80	3.29	<0.001	-44	-22	34	
0.584	0.420	87	0.120	0.583	0.757	4.73	3.89	<0.001	18	-8	-30	Right para-hippocampal gyrus
				0.915	0.757	4.15	3.53	<0.001	20	2	-34	
0.957	0.761	22	0.428	0.847	0.757	4.31	3.63	<0.001	-48	24	10	Left cerebral white matter
0.938	0.630	27	0.378	0.860	0.757	4.28	3.61	<0.001	54	6	-36	Right temporal pole
0.920	0.630	31	0.344	0.976	0.899	3.92	3.38	<0.001	54	12	10	Right opercular part of the inferior frontal gyrus
				0.995	0.899	3.71	3.24	0.001	64	16	16	
0.994	0.761	6	0.700	0.988	0.899	3.82	3.31	<0.001	12	-50	64	Right cerebral white matter
0.996	0.761	4	0.761	0.999	0.906	3.57	3.14	0.001	50	-6	-24	Right cerebral white matter
0.996	0.761	4	0.761	0.999	0.906	3.57	3.13	0.001	48	-16	-30	Right inferior temporal gyrus
0.998	1.000	2	0.842	0.999	1.000	3.55	3.12	0.001	-4	-18	-10	Left ventral DC

Figure 4A

Cluster-level				Peak-level					x	y	z	Brain region(s)
p FWE-corr	p FDR-corr	K_E	P unc	p FWE-corr	p FDR-corr	T	Z_E	p unc				
0.006	0.128	83	0.128	0.004	0.164	4.77	3.91	<0.001	18	-10	-30	Right para-hippocampal gyrus
				0.014	0.284	4.15	3.53	<0.001	20	2	-34	Right entorhinal area

SI Appendix, Figure S7A

Cluster-level				Peak-level					x	y	z	Brain region(s)
p FWE-corr	p FDR-corr	K_E	P unc	p FWE-corr	p FDR-corr	T	Z_E	p unc				
0.834	0.899	47	0.250	0.822	0.381	4.34	3.65	<0.001	18	-6	-30	Right para-hippocampal gyrus
0.029	0.074	370	0.004	0.834	0.381	4.32	3.64	<0.001	-18	-30	8	Left thalamus Proper
				0.847	0.381	4.30	3.62	<0.001	-26	-24	2	
0.998	0.899	1	0.899	0.999	0.993	3.53	3.11	0.001	26	-18	-30	Right para-hippocampal gyrus
0.998	0.899	1	0.899	0.999	0.993	3.53	3.11	0.001	-50	-16	-24	Left cerebral white matter
0.998	0.899	1	0.899	0.999	0.993	3.52	3.10	0.001	24	-22	-28	Right para-hippocampal gyrus
0.998	0.899	1	0.899	0.999	0.993	3.51	3.09	0.001	-58	-60	16	Left angular gyrus

Figure 4D

Cluster-level				Peak-level					x	y	z	Brain region(s)
p FWE-corr	p FDR-corr	K_E	P unc	p FWE-corr	p FDR-corr	T	Z_E	p unc				
0.008	0.156	73	0.156	0.006	0.194	4.54	3.77	<0.001	22	-18	-30	Right para-hippocampal gyrus
				0.010	0.194	4.34	3.65	<0.001	18	-6	-30	

SI Appendix, Figure S7C

Cluster-level				Peak-level					x	y	z	Brain region(s)
p FWE-corr	*p FDR-corr*	K_E	*P unc*	*p FWE-corr*	*p FDR-corr*	*T*	Z_E	*p unc*				
0.915	0.757	32	0.336	0.269	0.511	5.29	4.20	<0.001	14	-78	52	
0.988	0.762	10	0.605	0.759	0.593	4.46	3.73	<0.001	12	-22	24	Right lateral ventricle
0.721	0.757	65	0.174	0.837	0.593	4.33	3.64	<0.001	62	16	20	Right opercular part of the inferior frontal gyrus
				0.995	0.713	3.71	3.24	0.001	50	14	8	
0.982	0.757	13	0.550	0.918	0.680	4.15	3.53	<0.001	-44	-6	0	Left posterior insula
0.970	0.757	18	0.476	0.984	0.713	3.85	3.33	<0.001	-54	-8	28	Left precentral gyrus
0.994	0.762	6	0.700	0.984	0.713	3.85	3.33	<0.001	18	-26	22	Right lateral ventricle
0.993	0.762	7	0.673	0.987	0.713	3.82	3.31	<0.001	-30	-68	20	Left cerebral white matter
0.990	0.762	9	0.626	0.994	0.713	3.73	3.25	0.001	34	-18	-14	Right hippocampus
0.999	1.000	1	0.898	0.999	1.000	3.52	3.10	0.001	14	-26	16	Right lateral ventricle

Figure 4G

Cluster-level				Peak-level					x	y	z	Brain region(s)
p FWE-corr	*p FDR-corr*	K_E	*P unc*	*p FWE-corr*	*p FDR-corr*	*T*	Z_E	*p unc*				
0.892	0.842	37	0.288	0.779	0.978	4.46	3.72	<0.001	20	-12	-26	Right para-hippocampal gyrus
0.756	0.842	59	0.183	0.803	0.978	4.42	3.70	<0.001	54	0	50	Right precentral gyrus
0.998	0.894	2	0.837	0.960	0.978	4.03	3.45	<0.001	2	-58	50	Right precuneus
0.986	0.894	12	0.556	0.974	0.978	3.96	3.40	<0.001	20	-96	-16	
0.995	0.894	6	0.691	0.984	0.978	3.88	3.35	<0.001	-2	-58	50	Left precuneus
0.998	0.894	3	0.792	0.999	0.978	3.59	3.15	0.001	46	-26	-26	Right inferior temporal gyrus
0.998	0.894	2	0.837	0.999	0.978	3.58	3.15	0.001	4	-56	48	Right precuneus
0.995	0.894	6	0.691	0.999	0.978	3.55	3.12	0.001	64	0	34	
0.999	0.894	1	0.894	0.999	0.978	3.54	3.11	0.001	-20	-88	30	Left superior occipital gyrus
0.999	0.894	1	0.894	0.999	0.978	3.52	3.10	0.001	36	2	36	Right cerebral white matter

SI Appendix, Figure S8A

Cluster-level				Peak-level					x	y	z	Brain region(s)
p FWE-corr	*p FDR-corr*	K_E	*P unc*	*p FWE-corr*	*p FDR-corr*	*T*	Z_E	*p unc*				
0.045	0.567	12	0.567	0.051	0.647	3.73	3.25	0.001	34	-18	-14	Right hippocampus

SI Appendix, Figure S10B

Table S1. List of significant clusters from all source localisation analyses. This includes MNI co-ordinates (*x, y, z*) of the peak voxel, corresponding brain region, peak- and cluster- level family-wise error (*FWE*) and false discovery rate (*FDR*) corrected p-values, cluster size (K_E), t-statistic (*T*), z-statistic (Z_E) and uncorrected (*unc*) p-values for all clusters that pass a threshold of p<0.001 (uncorrected). Results reported in the main text are highlighted in orange.
